# (*N*,*N*-Di­allyl­dithio­carbamato-κ^2^ *S*,*S*′)tri­phenyltin(IV) and bis­(*N*,*N*-di­allyl­dithio­carbamato-κ^2^ *S*,*S*′)di­phenyl­tin(IV): crystal structure, Hirshfeld surface analysis and computational study

**DOI:** 10.1107/S2056989020000122

**Published:** 2020-01-10

**Authors:** Farah Natasha Haezam, Normah Awang, Nurul Farahana Kamaludin, Mukesh M. Jotani, Edward R. T. Tiekink

**Affiliations:** a Environmental Health and Industrial Safety Programme, Faculty of Health Sciences, Universiti Kebangsaan Malaysia, Jalan Raja Muda Abdul Aziz, 50300 Kuala Lumpur, Malaysia; b Department of Physics, Bhavan’s Sheth R. A. College of Science, Ahmedabad, Gujarat 380001, India; c Research Centre for Crystalline Materials, School of Science and Technology, Sunway University, 47500 Bandar Sunway, Selangor Darul Ehsan, Malaysia

**Keywords:** crystal structure, organotin, di­thio­carbamate, Hirshfeld surface analysis, computational chemistry

## Abstract

The crystal and mol­ecular structures of the title organotin di­thio­carbamate compounds, [Sn(C_6_H_5_)_3_(C_7_H_10_NS_2_)] (I) and [Sn(C_6_H_5_)_2_(C_7_H_10_NS_2_)_2_] (II), present very distinct tin atom coordination geometries. In (I), the di­thio­carbamate ligand is asymmetrically coordinating with the resulting C_3_S_2_ donor set defining a coordination geometry inter­mediate between square-pyramidal and trigonal–bipyramidal. In (II), two independent mol­ecules comprise the asymmetric unit, which differ in the conformations of the allyl substituents and in the relative orientations of the tin-bound phenyl rings. The di­thio­carbamate ligands in (II) coordinate in an asymmetric mode but the Sn—S bonds are more symmetric than observed in (I). The resulting C_2_S_4_ donor set approximates an octa­hedral coordination geometry with a *cis*-disposition of the *ipso*-carbon atoms and with the more tightly bound sulfur atoms approximately *trans*. The only directional inter­molecular contacts in the crystals of (I) and (II) are of the type phenyl-C—H⋯π(phen­yl) and vinyl­idene-C—H⋯π(phen­yl), respectively, with each leading to a supra­molecular chain propagating along the *a-*axis direction. The calculated Hirshfeld surfaces emphasize the importance of H⋯H contacts in the crystal of (I), *i.e*. contributing 62.2% to the overall surface. The only other two significant contacts also involve hydrogen, *i.e*. C⋯H/H⋯C (28.4%) and S⋯H/H⋯S (8.6%). Similar observations pertain to the individual mol­ecules of (II), which are clearly distinguishable in their surface contacts, with H⋯H being clearly dominant (59.9 and 64.9%, respectively) along with C⋯H/H⋯C (24.3 and 20.1%) and S⋯H/H⋯S (14.4 and 13.6%) contacts. The calculations of energies of inter­action suggest dispersive forces make a significant contribution to the stabilization of the crystals. The exception is for the C—H⋯π contacts in (II) where, in addition to the dispersive contribution, significant contributions are made by the electrostatic forces.

## Abstract 2

Distinct tin coordination geometries are found in the title compounds, *i.e*. (C_6_H_5_)_3_Sn[S_2_CN(CH_2_C(H)=CH_2_)_2_] has a geometry inter­mediate between square-pyramidal and trigonal–bipyramidal, while the geometry for (C_6_H_5_)_2_Sn[S_2_CN(CH_2_C(H)=CH_2_)_2_]_2_ is based on an octa­hedron with *cis*-phenyl groups.

## Chemical context   

Di­thio­carbamate anions of general formula ^−^S_2_CN*RR*′, *R*/*R*′ = H, alkyl and aryl, are readily prepared from the facile reaction of an amine with CS_2_ in the presence of base. Thus, the number of derivatives which can be prepared is largely dictated by the availability of amines and hence, an enormous range of di­thio­carbamate anions are available for complexation to metals/heavy elements. A key inter­est in developing metal/heavy element compounds of di­thio­carbamates relates to their biological potential (Hogarth, 2012 ▸). In the context of anti-cancer properties, a number of recent reports have described the efficacy of phosphanegold (Jamaludin *et al.*, 2013 ▸), zinc (Tan *et al.*, 2015 ▸) and bis­muth (Ishak *et al.*, 2014 ▸) di­thio­carbamates, buoyed by the observation that many of these species promote cancer cell death by apoptosis; bis­muth derivatives exhibit *in vivo* anti-tumour activity (Li *et al.*, 2007 ▸). Organotin compounds are well known for their anti-cancer potential (Gielen & Tiekink, 2005 ▸) and there is a strong body of literature on organotin di­thio­carbamates in this context (Tiekink, 2008 ▸).
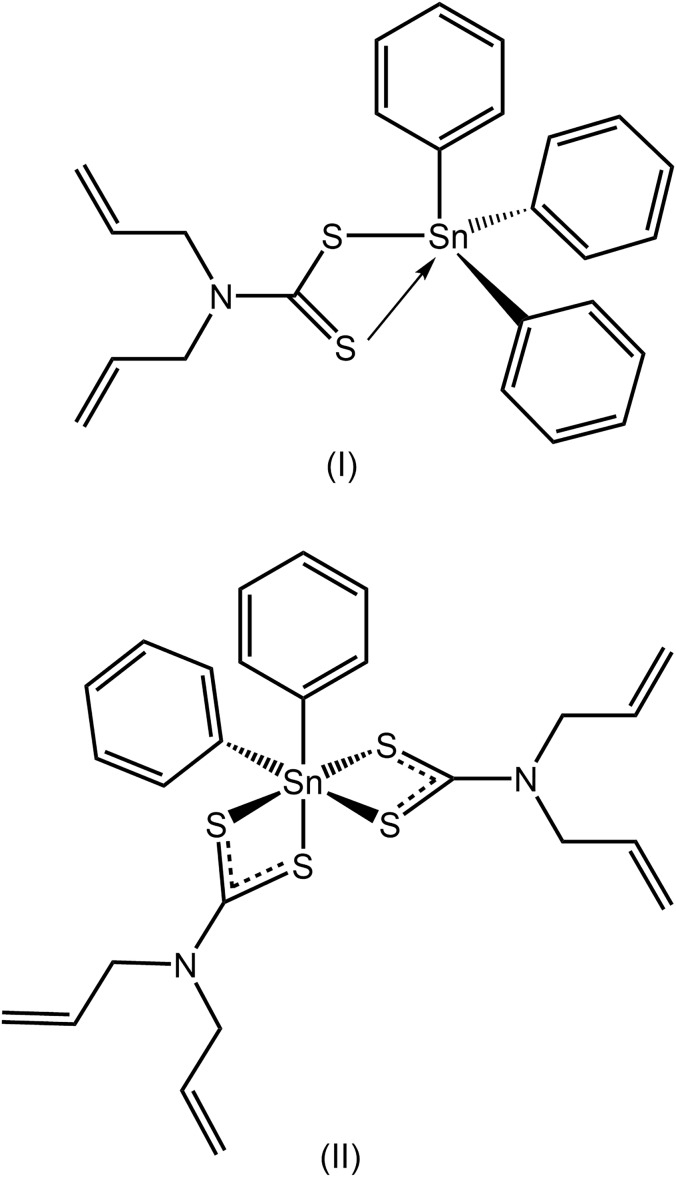


In the past few years, there has been a resurgence of inter­est in the anti-cancer activity of organotin di­thio­carbamates (Khan *et al.*, 2015 ▸; Mohamad, Awang, Kamaludin *et al.*, 2016 ▸) and very recently, a report on the *in vitro* cytotoxicity trial of several tin di­allyl­dithio­carbamate compounds was described as well as a preliminary assessment of anti-microbial activity (Adeyemi *et al.*, 2019 ▸); some phosphane-gold(I) and phosphane-silver(I) di­thio­carbamates are known to be bactericidal based on pharmacokinetic studies (Sim *et al.*, 2014 ▸; Tan, Tan *et al.*, 2019 ▸). The aforementioned report on tin di­allyl­dithio­carbamate compounds (Adeyemi *et al.*, 2019 ▸) also presented the first crystal-structure determinations for tin compounds of di­allyl­dithio­carbamate. In a continuation of recent structural studies in this area (Mohamad *et al.*, 2017 ▸, 2018*a* ▸,*b* ▸; Haezam *et al.*, 2019 ▸), herein, two organotin compounds of di­allyl­dithio­carbamate, (C_6_H_5_)_3_Sn[S_2_CN(CH_2_C(H)=CH_2_)_2_], (I), and (C_6_H_5_)_2_Sn[S_2_CN(CH_2_C(H)=CH_2_)_2_]_2_, (II), have been synth­esized and studied by X-ray crystallography. In addition, the supra­molecular associations in their crystals have been evaluated by Hirshfeld surface analyses and computational chemistry.

## Structural commentary   

**Figure 1:**
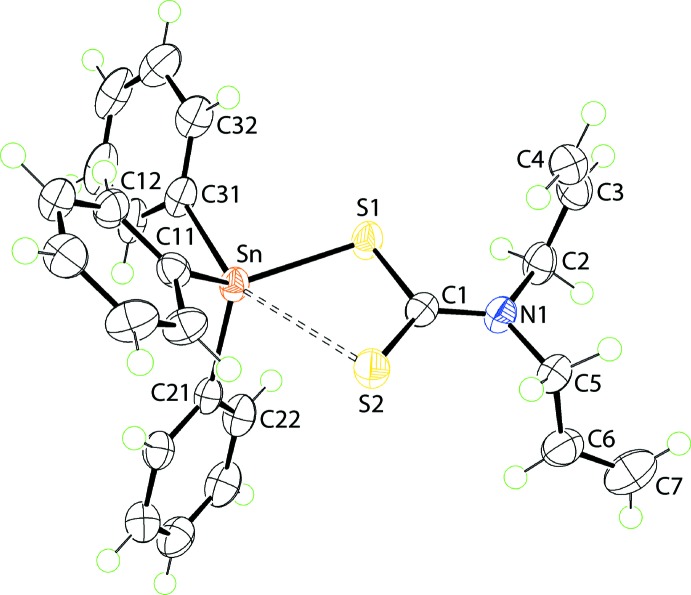
The mol­ecular structure of (I) showing the atom-labelling scheme and displacement ellipsoids at the 70% probability level.

The tin atom in (I), Fig. 1 ▸, is coordinated by three *ipso*-carbon atoms of the phenyl groups as well as by an asymmetrically bound di­thio­carbamate anion, Table 1 ▸. There is a relatively large disparity in the Sn—S separations, *i.e*. Δ(Sn—S) = [(Sn—S_long_) - (Sn—S_short_)] = 0.47 Å, indicating that the Sn—S2 inter­action is weak. Evidence in support of this conclusion is seen in the pattern of C—S bond lengths. Thus, the C1—S2 bond involving the less tightly bound S2 atom is about 0.07 Å shorter than the analogous bond with the tightly bound S1 atom. Nevertheless, there is a clear influence exerted by the S2 atom upon the Sn—C bond lengths with the Sn—C31 bond being appreciably longer than the other Sn—C bonds. This is traced to the *trans* effect exerted by the S2 atom as this forms a S2—Sn—C31 angle 156.01 (5)°. It is noted that there is no other (approximate) *trans* angle subtended at the tin atom in (I). Assuming a five-coordinate, C_3_S_2_, geometry, the range of angles subtended at the tin atom is 65.470 (14)°, for the S1—Sn—S2 chelate angle, to the aforementioned *trans* angle. The value of τ is a convenient descriptor for the assignment of a five-coordinate geometry, which ranges in value from 0.0 for an ideal square pyramid to 1.0 for an ideal trigonal bipyramid (Addison *et al.*, 1984 ▸). The value of τ the case of (I) is 0.45, which is indicative of an inter­mediate geometry with a slight tendency towards square pyramidal. On the other hand, should the coordination geometry be considered C_3_S tetra­hedral, *i.e*. the weak Sn—S2 bond was ignored, the range of tetra­hedral angles would be 91.01 (5)°, for S1—Sn—C31, to 128.76 (5)°, for S1—Sn—C11. Finally, it is noted the C1—N1 bond length of 1.330 (3) Å is consistent with significant double-bond character in this bond, which arises from a major contribution of the ^2−^S_2_C=N^+^(CH_2_C(H)=CH_2_)_2_ canonical form to the electronic structure of the di­thio­carbamate ligand.

**Figure 2:**
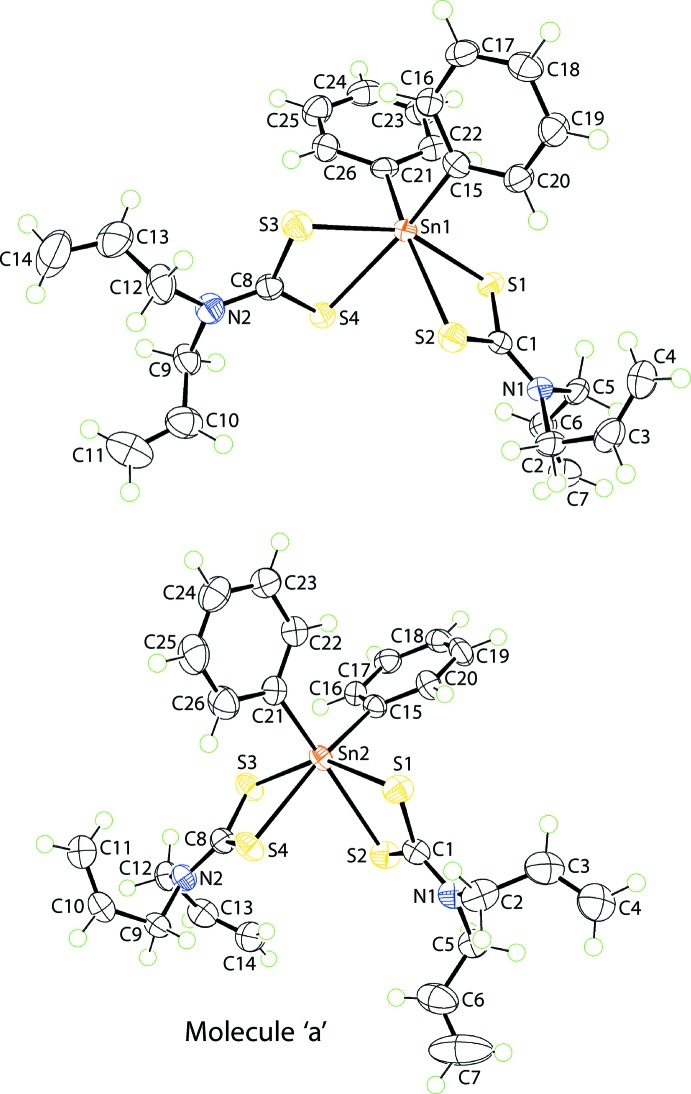
The mol­ecular structures of the two independent mol­ecules comprising the asymmetric unit of (II) showing the atom-labelling scheme and displacement ellipsoids at the 70% probability level.

A distinct coordination geometry for the tin atoms is noted for (II), Fig. 2 ▸, for which two independent mol­ecules comprise the crystallographic asymmetric unit. The tin atom in each mol­ecule is coordinated by two *ipso*-carbon atoms of the phenyl groups as well as by two asymmetrically bound di­thio­carbamate anions, Table 2 ▸. There is a disparity in the Sn—S separations, *i.e*. Δ(Sn—S) = 0.19 and 0.11 Å, for the S1- and S3-di­thio­carbamate anions of the first independent mol­ecule; the comparable values for the second mol­ecule are similar at 0.21 and 0.11 Å. The disparities in Δ(Sn—S) are reflected in the associated C—S bond distances, Table 2 ▸. Gratifyingly, the greater differences in C—S bonds, *i.e*. 0.03 and 0.04 Å for the S1-di­thio­carbamate anions of each independent mol­ecule, are correlated with the greater values in Δ(Sn—S). The C1—N1 and C8—N2 bond lengths in both mol­ecules are short for the reasons mentioned for (I) above. The C_2_S_4_ coordination geometry is based on an octa­hedron and has a *cis*-disposition of the *ipso*-carbon atoms with the more tightly bound sulfur atoms close to being *trans*. A partial explanation of the lengthening of the Sn—S2 and Sn—S4 bonds relates to the *trans*-influence exerted by the phenyl substituents approximately opposite the S2 and S4 atoms.

**Figure 3:**
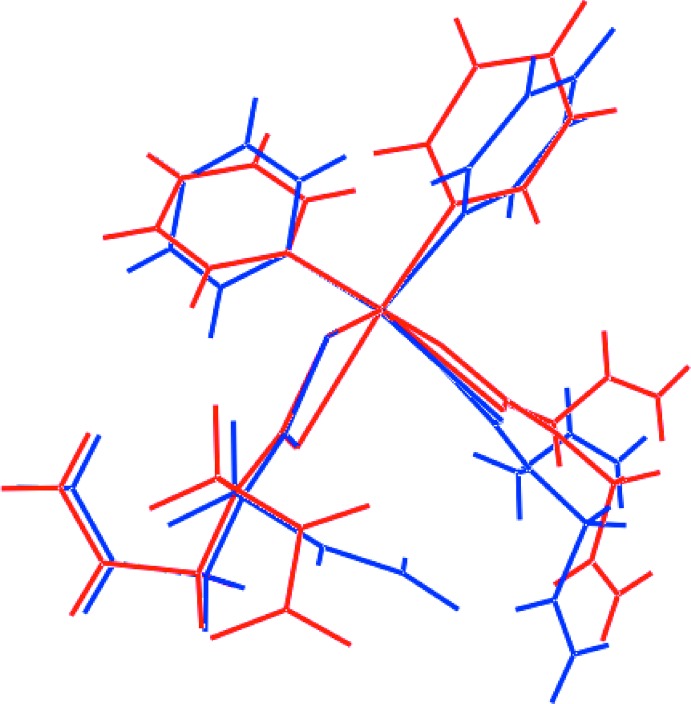
Overlay diagram of the independent mol­ecules comprising the asymmetric unit of (II): (*a*) Sn1-containing mol­ecule (red image) and (*b*) inverted-Sn2-mol­ecule (blue). The mol­ecules are overlapped so that the (Sn1,S1,S2,C1) and (Sn2,S3,S4,C8) residues are coincident.

A view of the superimposition of the two mol­ecules comprising the asymmetric unit in (II) is shown in Fig. 3 ▸ whereby the Sn1- and inverted-Sn2-mol­ecules are overlapped so that two chelate rings, *i.e*. (Sn1,S1,S2,C1) and (Sn2,S3,S4,C8), are coincident. This shows there are non-trivial conformational differences between the mol­ecules. While the dihedral angles between the two phenyl substituents are equal within experimental error in the two mol­ecules, *i.e*. 81.28 (13) and 81.63 (14)°, more telling are the angles they form with the respective, *cis*-disposed chelate rings, *i.e*. 81.06 (10) and 35.93 (10)° for the Sn1-mol­ecule and 15.35 (11) and 74.71 (6)° for the Sn2-mol­ecule. Differences are also noted in the relative orientations of the allyl substituents. Thus, for the overlapped di­thio­carbamate ligands, the N1—C5—C6—C7 torsion angle of −122.3 (3)° is an outlier with respect to the other torsion angles with the direct equivalent angle for the inverted Sn2-mol­ecule being 13.3 (4)°. While the N—C—C—C torsion angles for the second pair of di­thio­carbamate ligands are similar, Table 2 ▸, there is a misalignment of these ligands as seen in the dihedral angle formed between the chelate rings of 80.98 (5) and 76.55 (6)° for the Sn1- and Sn2-mol­ecules, respectively.

The difference in coordination modes of the di­thio­carbamate ligands and coordination geometries are related, at least in part, to the different Lewis acid strength of the tin atoms, with the Lewis acidity in the tri­phenyl­tin species being significantly less than that in the di­phenyl­tin species.

## Supra­molecular features   

**Figure 4:**
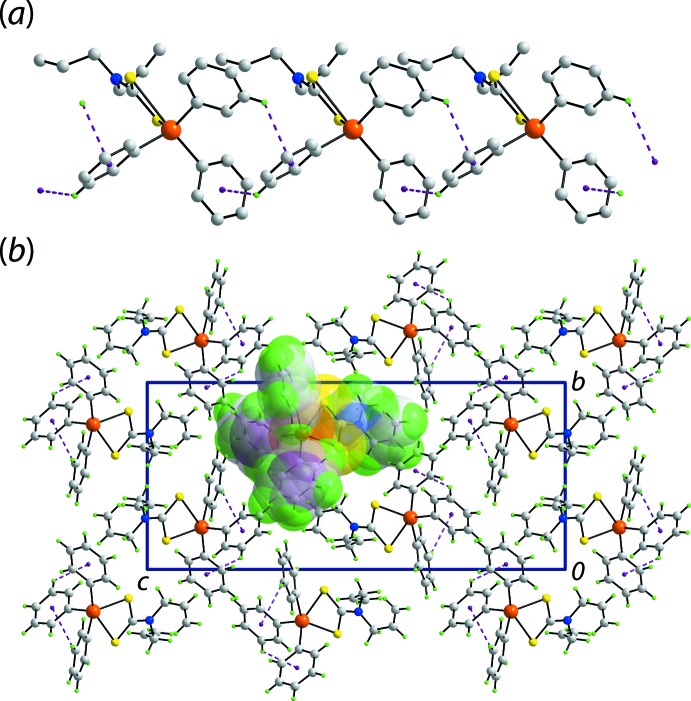
Mol­ecular packing in the crystal of (I): (*a*) supra­molecular chain along the *a*-axis direction sustained by phenyl-C—H⋯π(phen­yl) inter­actions shown as purple dashed lines (non-participating hydrogen atoms have been removed) and (*b*) a view of the unit-cell contents in projection down the *a* axis with one chain highlighted in space-filling mode.

The only directional point of contact between mol­ecules based on the distance criteria in *PLATON* (Spek, 2020 ▸) are phenyl-C—H⋯π(phen­yl) inter­actions, Table 3 ▸. Here, the (C21–C26) ring is pivotal by donating a C—H atom to a symmetry-related (C31–C36) ring and the same time accepting a phenyl-C—H⋯π(phen­yl) inter­action from a (C11–C16) ring to construct a linear, supra­molecular chain aligned along the *a*-axis direction, Fig. 4 ▸(*a*). The chains assemble in the crystal without directional inter­actions between them, Fig. 4 ▸(*b*).

**Figure 5:**
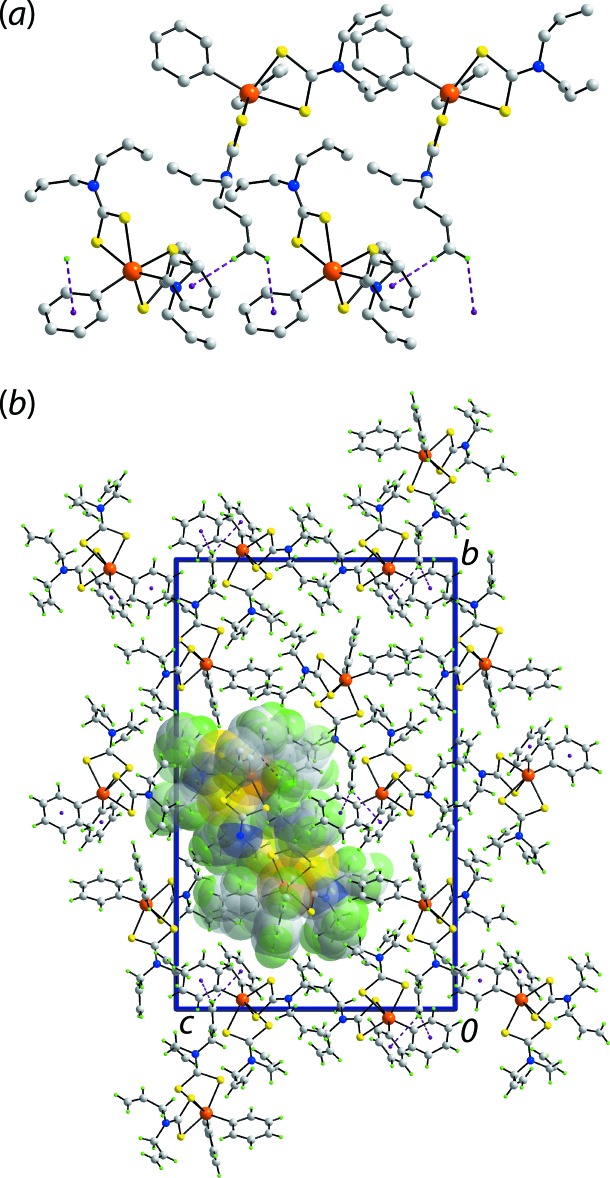
Mol­ecular packing in the crystal of (II): (*a*) supra­molecular chain along the *a*-axis direction sustained by methyl­ene-C—H⋯π(phen­yl) inter­actions shown as purple dashed lines (non-participating hydrogen atoms have been removed) and (*b*) a view of the unit-cell contents in projection down the *a* axis with one chain highlighted in space-filling mode.

The mol­ecular packing in (II) is also largely devoid of directional inter­actions. Indeed, the only connections evident are vinyl­idene-C—H⋯π(phen­yl) inter­actions, Table 4 ▸, which serve to link the independent mol­ecules comprising the asymmetric unit into a supra­molecular chain aligned along the *a-*axis direction. In essence, the vinyl­idene-hydrogen atoms of the Sn1-mol­ecule bridge translationally related Sn2-mol­ecules into a linear chain, Fig. 5 ▸(*a*). The chains pack without directional inter­actions between them, Fig. 5 ▸(*b*).

## Hirshfeld surface analysis   

**Figure 6:**
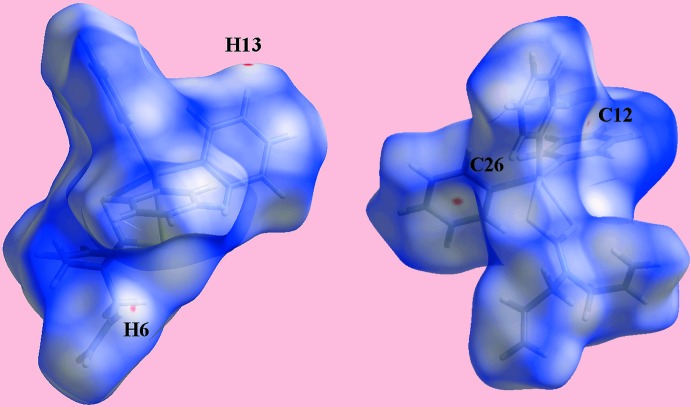
Two views of Hirshfeld surface for (I) mapped over *d*_norm_ in the range −0.028 to +1.257 arbitrary units.

**Figure 8:**
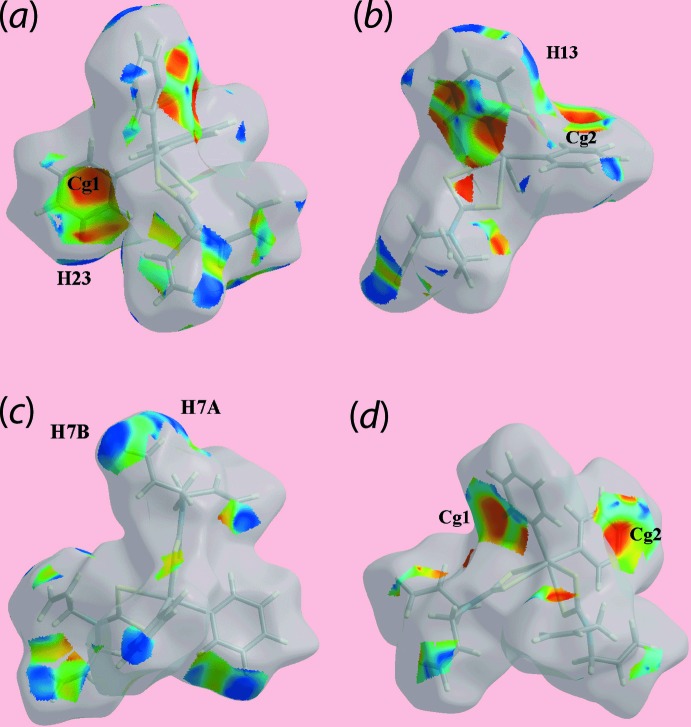
Views of Hirshfeld surfaces mapped with the shape-index property highlighting donors and acceptors of the inter­molecular C—H⋯π contacts for (*a*) and (*b*) (I), and (*c*) and (*d*) (II).

**Figure 9:**
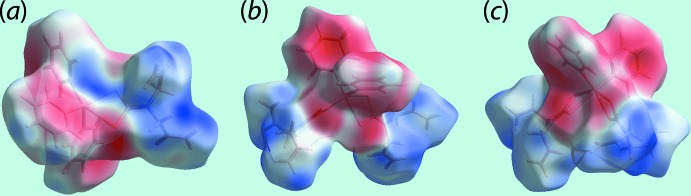
Views of Hirshfeld surfaces mapped over the electrostatic potential (the red and blue regions represent negative and positive electrostatic potentials, respectively) for (*a*) (I) in the range −0.032 to +0.043 atomic units (a.u.), (*b*) the Sn1-mol­ecule in (II) in the range −0.039 to +0.040 a.u. and (*c*) the Sn2-mol­ecule in (II) in the range −0.038 to +0.047 a.u.

In order to gain further insight into the mol­ecular packing of each of (I) and (II), Hirshfeld surface calculations were performed with *Crystal Explorer 17* (Turner *et al.*, 2017 ▸) following literature protocols (Tan, Jotani *et al.*, 2019 ▸). The calculations highlight the influence of the discussed C—H⋯π inter­actions (Tables 3 ▸ and 4 ▸) as well the short inter­atomic contacts collated in Table 5 ▸. The short inter­atomic contacts are indicated as diminutive or faint-red spots near the participating atoms on the Hirshfeld surfaces mapped over *d*_norm_ for (I) and (II) in Figs. 6 ▸ and 7 ▸, respectively. Further, the donors and acceptors of the inter­molecular C—H⋯π contacts for both (I) and (II) are evident as the blue bumps and red concave regions, respectively, on the Hirshfeld surfaces mapped with shape-index property shown in Fig. 8 ▸. In the absence of potential hydrogen bonds in (I) and (II), both the blue and red regions corresponding to positive and negative electrostatic potential, respectively, on Hirshfeld surfaces mapped over electrostatic potential in Fig. 9 ▸ and arise owing to the polarization of charges towards the participating residues.

**Figure 10:**
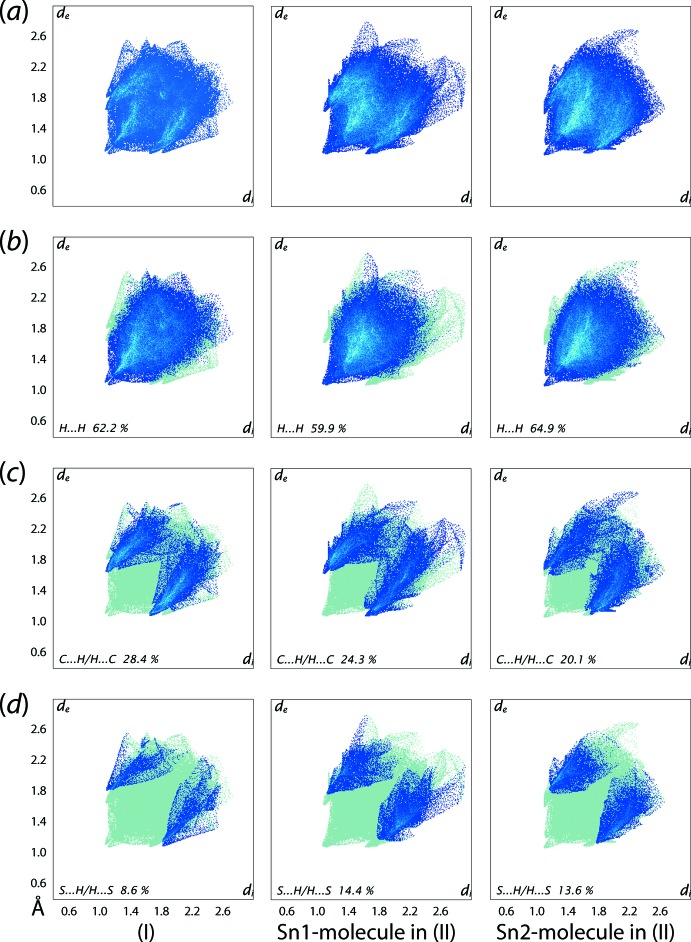
(*a*) A comparison of the full two-dimensional fingerprint plot for (I) and for the Sn1- and Sn2-mol­ecules of (II), and those delineated into (*b*) H⋯H, (*c*) C⋯H/H⋯C and (*d*) S⋯H/H⋯S contacts.

The overall two-dimensional fingerprint plots for (I) and the individual mol­ecules of (II) are illustrated in Fig. 10 ▸(*a*), and those delineated into H⋯H, C⋯H/H⋯C and S⋯H/H⋯S contacts are illustrated in Fig. 10 ▸(*b*)–(*d*), respectively. The percentage contributions from different atom–atom contacts to the Hirshfeld surfaces of (I), Sn1- and Sn2-mol­ecules of (II) are qu­anti­tatively summarized in Table 6 ▸. In the fingerprint plot delineated into H⋯H contacts for (I), Fig. 10 ▸(*b*), a pair of small and proximate peaks at *d*_e_ + *d*_i_ ∼2.2 Å results from the presence of a short inter­atomic contact between the phenyl-H15 and H24 atoms, Table 5 ▸. The presence of a single peak at *d*_e_ + *d*_i_ ∼2.2 Å in the analogous plot for the Sn1-mol­ecule of (II) is due to the short H⋯H contact between the phenyl-H17 and H26 atoms. Another short inter­atomic H⋯H contact involving the H17 and H18*A* atoms of the Sn1-mol­ecule and the H9*A*2 and H13*A* atoms of the Sn2-mol­ecule, Table 5 ▸, are evident as the pair of peaks at *d*_e_ + *d*_i_ ∼2.2 Å and at *d*_e_ + *d*_i_ ∼2.3 Å in the corresponding delineated plot for the Sn2-mol­ecule.

The presence of short inter­atomic C⋯H/H⋯C contacts in each of (I) and (II), summarized in Table 5 ▸, are evident as the forceps-like tips at *d*_e_ + *d*_i_ ∼2.8 Å in Fig. 10 ▸(*a*). Also, the inter­molecular C—H⋯π contacts are characterized as a pair of wings in their respective delineated plots shown in Fig. 10 ▸(*c*). The short inter­atomic C⋯H/H⋯C contacts in the crystal of (II) appear as a pair of forceps-like tips at *d*_e_ + *d*_i_ ∼2.7 Å for the Sn1-mol­ecule and as two pairs of similar adjoining tips at the same distances *d*_e_ + *d*_i_ ∼2.8 Å for the Sn2-mol­ecule in the plots of Fig. 10 ▸(*c*). For (I), in the fingerprint plot delineated into S⋯H/H⋯S contacts of Fig. 10 ▸(*d*), the short inter­atomic contacts involving thio­carbamate-S1 and the H2*A* and H7*B* atoms are evident as the pair of conical tips at *d*_e_ + *d*_i_ ∼2.9 Å. Similar contacts in the crystal of (II) are also evident as the conical tips at *d*_e_ + *d*_i_ ∼2.9 Å in Fig. 10 ▸(*d*) in the upper and lower regions of the plots for the Sn1- and Sn2-mol­ecules, respectively.

## Computational chemistry   

The pairwise inter­action energies between the mol­ecules within the crystals of (I) and (II) were calculated by summing up four energy components, namely the electrostatic (*E*_ele_), polarization (*E*_pol_), dispersion (*E*_dis_) and exchange-repulsion (*E*_rep_) energies, in accord with literature protocols (Turner *et al.*, 2017 ▸). In the present analysis, these energies were obtained by using the wave function calculated at the HF/3-21G level of theory. The specific contacts and associated energies are qu­anti­tatively summarized in Table 7 ▸. An analysis of these energies for (I) and (II) reveals that the dispersive component makes the major contribution to all the specified inter­molecular inter­actions in the crystals of (I) and (II). However, as clearly evident from the relevant inter­action energies listed in Table 7 ▸ and in the Hirshfeld surfaces mapped over the electrostatic potential of Fig. 9 ▸, where intense blue and red regions are apparent around the donors and acceptors, the C—H⋯π contacts in (II) have more significant contributions from the *E*_ele_ component, in contrast to mainly dispersive contributions in the case of (I).

**Figure 11:**
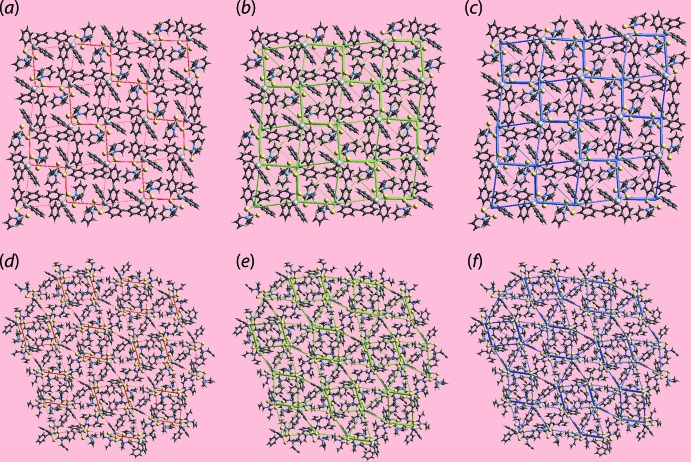
The energy frameworks calculated for (I) viewed down the *a*-axis direction showing the (*a*) electrostatic potential force, (*b*) dispersion force and (*c*) total energy. The corresponding plots for (II), viewed down the *a*-axis direction are shown in (*d*)–(*f*), respectively. The energy frameworks were adjusted to the same scale factor of 50 with a cut-off value of 5 kJ mol^−1^ within 2 × 2 × 2 unit cells.

A further noticeable observation about the strength of the inter­molecular inter­actions from Table 7 ▸ is that those inter­molecular contacts arising from the same pair of symmetry-related mol­ecules have the greater inter­action energies. The magnitudes of inter­molecular energies were also represented graphically by energy frameworks to view the supra­molecular architecture of both the crystals through cylinders joining the centroids of mol­ecular pairs using red, green and blue colour codes for the *E*_ele_, *E*_disp_ and *E*_tot_ terms, respectively. In summary, the images of Fig. 11 ▸ highlight the importance of dispersion forces in the crystals of (I) and (II).

## Database survey   

As a result of having several important applications, such as biological activity as alluded to in the *Chemical Context*, a relatively large number of organotin di­thio­carbamates have been synthesized and investigated by X-ray crystallography (Tiekink, 2008 ▸). The coordination geometry described for (I) conforms with literature expectations in that all *R*_3_Sn(S_2_CN*R*′*R*′′) mol­ecules conform to this structural motif (Tiekink, 2008 ▸; Mohamad *et al.*, 2018*a* ▸). The Sn—S1 bond length in (I) of 2.4749 (4) Å is slightly longer that the average Sn—S_short_ bond of 2.47 Å in all Ph_3_Sn(S_2_CN*R*′*R*′′) structures, while the Sn—S2 bond of 2.9456 (5) Å in (I) is about 0.10 Å shorter than the average Sn—S_long_ of 3.04 Å in these structures. Consistent with these trends, Δ(Sn—S) in (I) of 0.47 Å is less than the average Δ(Sn—S) value of 0.57 Å calculated from all Ph_3_Sn(S_2_CN*R*′*R*′′) structures.

Greater structural diversity is noted for *R*_2_Sn(S_2_CN*R*′*R*′′)_2_ (Tiekink, 2008 ▸), including differences in coordination numbers and geometries (Mohamad, Awang, Jotani *et al.*, 2016 ▸). Of the now, 17 structures of the general formula Ph_2_Sn(S_2_CN*R*′*R*′′)_2_, nine adopt the *cis*-C_2_S_4_ structural motif exemplified by (II), including the two polymorphs of Ph_2_Sn(S_2_CNEt_2_)_2_ (Lindley & Carr, 1974 ▸; Hook *et al.*, 1994 ▸). The remaining structures adopt the usual motif for *R*_2_Sn(S_2_CN*R*′*R*′′)_2_, namely a geometry based on a bipyramidal skewed-bipyramid. Here, the di­thio­carbamate ligands coordinate in an asymmetric fashion with the tin-bound phenyl substituents disposed to lie over the weaker Sn—S bonds, exemplified by the two independent mol­ecules comprising the asymmetric unit of Ph_2_Sn[S_2_CN(Me)Hex]_2_ (Hex = *n*-hexyl, –C_7_H_15_) (Ramasamy *et al.*, 2013 ▸). Clearly there is a subtle inter­play between the electronic and steric characteristics of the di­thio­carbamate ligands and mol­ecular packing effects in determining the structural motif adopted by Ph_2_Sn(S_2_CN*R*′*R*′′)_2_ in their respective crystals.

## Synthesis and crystallization   

All chemicals and solvents were used as purchased without purification. The melting point was determined using an automated melting point apparatus (MPA 120 EZ-Melt). Carbon, hydrogen and nitro­gen analyses were performed on a Leco CHNS-932 Elemental Analyzer.

The synthesis of (I) and (II) followed established literature procedures (Awang *et al.*, 2011 ▸; Ajibade *et al.*, 2011 ▸). For each synthesis, initially, di­allyl­amine (Aldrich; 1.27 ml, 10 mmol) dissolved in ethanol (30 ml) was stirred under ice-bath conditions for 20 mins. A 25% ammonia solution (1 to 2 ml) was added followed by stirring for 30 mins to establish basic conditions. Then, a cold ethano­lic solution of carbon di­sulfide (0.60 ml, 10 mmol) was added dropwise into the solution and stirred for about 2 h.

For (I), tri­phenyl­tin(IV) dichloride (Merck; 3.85 g, 10 mmol) dissolved in ethanol (20–30 ml) was added dropwise into the di­allyl­dithio­carbamate solution and further stirred for 2 to 3 h. Next, the precipitate that formed was filtered off and washed with cold ethanol a few times to remove any impurities. Finally, the filtered precipitate was dried in a desiccator overnight. Recrystallization was carried out by dissolving the compound in a chloro­form and ethanol solvent mixture (5 ml; 1:1 *v*/*v*), which was allowed to slowly evaporate at room temperature yielding colourless crystals. Yield: 44%. M.p. 454.8–456.2 K. Elemental analysis: calculated (%): C 57.51, H 4.79, N 2.68. Found (%): C 56.92, H 4.93, N 2.93.

Compound (II) was prepared and recrystallized as for (I) but, using di­phenyl­tin(IV) dichloride (Merck; 1.72 g, 5 mmol) dissolved in ethanol (20-30 ml). Yield: 52%. M.p. 332.5–334.4 K. Elemental analysis: calculated (%): C 50.59, H 4.86, N 4.54. Found (%): C 50.20, H 4.80, N 4.20.

## Refinement   

Crystal data, data collection and structure refinement details are summarized in Table 8 ▸. Carbon-bound H atoms were placed in calculated positions (C—H = 0.95–0.99 Å) and were included in the refinement in the riding-model approximation, with *U*_iso_(H) set to 1.2*U*_eq_(C). In (II), the maximum and minimum residual electron density peaks of 1.23 and 0.73 e Å^−3^, respectively, were located 0.80 and 0.74 Å from the S2 and Sn1 atoms, respectively.

## Supplementary Material

Crystal structure: contains datablock(s) I, II, global. DOI: 10.1107/S2056989020000122/hb7884sup1.cif

Structure factors: contains datablock(s) I. DOI: 10.1107/S2056989020000122/hb7884Isup2.hkl

Structure factors: contains datablock(s) II. DOI: 10.1107/S2056989020000122/hb7884IIsup3.hkl

CCDC references: 1975797, 1975796

Additional supporting information:  crystallographic information; 3D view; checkCIF report

## Figures and Tables

**Figure 7:**
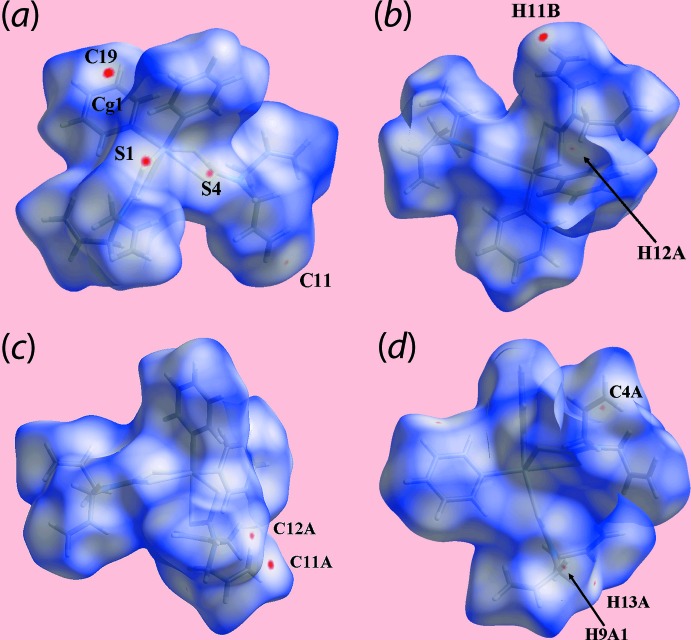
Views of the Hirshfeld surfaces for (II) mapped over *d*_norm_ for the (*a*) and (*b*) Sn1-mol­ecule in the range −0.026 to +1.372 arbitrary units, and (*c*) and (*d*) Sn2-mol­ecule in the range −0.027 to +1.383 arbitrary units.

**Table 1:** Selected geometric parameters (Å, °) for (I)

Parameter	(I)	Parameter	(I)
Sn—S1	2.4749 (4)	Sn—S2	2.9456 (5)
Sn—C11	2.1427 (19)	Sn—C21	2.130 (2)
Sn—C31	2.1673 (19)	C1—S1	1.7559 (19)
C1—S2	1.6894 (19)	C1—N1	1.330 (3)
			
S1—Sn—S2	65.470 (14)	C11—Sn—C21	111.15 (7)
C11—Sn—C31	104.14 (7)	C21—Sn—C31	107.13 (7)
S1—Sn—C11	128.76 (5)	S2—Sn—C31	156.01 (5)

**Table 2:** Selected geometric parameters (Å, °) for the two independent mol­ecules in (II)

Parameter	Sn1-mol­ecule	Sn2-mol­ecule
Sn—S1	2.5501 (6)	2.5585 (7)
Sn—S2	2.7393 (7)	2.7664 (7)
Sn—S3	2.5726 (7)	2.5700 (6)
Sn—S4	2.6754 (6)	2.6750 (6)
C1—S1	1.742 (3)	1.740 (3)
C1—S2	1.710 (3)	1.704 (3)
C8—S3	1.738 (3)	1.733 (3)
C8—S4	1.715 (3)	1.716 (3)
C1—N1	1.326 (3)	1.328 (4)
C8—N2	1.318 (3)	1.327 (3)
		
S1—Sn—S2	67.824 (19)	67.18 (2)
S3—Sn—S4	67.97 (2)	68.69 (2)
S1—Sn—S3	154.38 (2)	149.00 (2)
S2—Sn—C21	159.42 (7)	161.40 (7)
S4—Sn—C15	160.59 (7)	159.91 (7)
C15—Sn—C21	99.84 (9)	103.34 (9)
		
N1—C2—C3—C4	12.5 (4)	9.9 (4)^*a*^
N1—C5—C6—C7	−122.3 (3)	13.3 (4)^*a*^
N2—C9—C10—C11	105.3 (3)	105.2 (3)^*a*^
N2—C12—C13—C14	110.9 (4)	114.6 (4)^*a*^

Note: (*a*) for ease of comparison, the torsion angles are for the inverted Sn2-mol­ecule.

**Table 3:** Hydrogen-bond geometry (Å, °) for (I) *Cg*1 and *Cg*2 are the centroids of the (C21–C26) and (C31–C36) rings, respectively.

*D*—H⋯*A*	*D*—H	H⋯*A*	*D*⋯*A*	*D*—H⋯*A*
C13—H13⋯*Cg*1^i^	0.95	2.92	3.605 (2)	130
C23—H23⋯*Cg*2^ii^	0.95	2.99	3.720 (3)	134

Symmetry codes: (i) 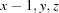; (ii) 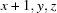.

**Table 4:** Hydrogen-bond geometry (Å, °) for (II) *Cg*1 and *Cg*2 are the centroids of the (C15*A*–C20*A*) and (C21*A*–C26*A*) rings, respectively.

*D*—H⋯*A*	*D*—H	H⋯*A*	*D*⋯*A*	*D*—H⋯*A*
C7—H7*A*⋯*Cg*1^i^	0.95	2.83	3.774 (3)	172
C7—H7*B*⋯*Cg*2^ii^	0.95	2.92	3.582 (3)	128

Symmetry codes: (i) 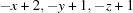; (ii) 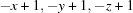.

**Table 5:** Summary of short inter­atomic contacts (Å) in (I) and (II)^*a*^

Contact	Distance	Symmetry operation
(I)		
C12⋯H6	2.76	−1 + *x*, *y*, *z*
C16⋯H5*B*	2.80	1 − *x*, − *y*, 1 − *z*
C25⋯H4*B*	2.78	*x*, 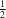 − *y*, − 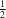 + *z*
C26⋯H13	2.74	1 + *x*, *y*, *z*
C33⋯H2*B*	2.79	1 − *x*, 1 − *y*, 1 − *z*
H15⋯H24	2.19	1 − *x*, − 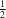 + *y*, 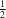 − *z*
S1⋯H7*B*	2.91	−1 + *x*, *y*, *z*
S1⋯H2*A*	2.96	1 − *x*, 1 − *y*, 1 − *z*
(II)		
S1⋯C11*A*	3.455 (3)	1 − *x*, 1 − *y*, 1 − *z*
S4⋯C12*A*	3.473 (3)	1 − *x*, 1 − *y*, 1 − *z*
C11⋯C23*A*	3.386 (5)	*x*, *y*, *z*
C11⋯H23*A*	2.81	*x*, *y*, *z*
C19⋯H11*B*	2.71	1 + *x*, 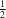 − *y*, 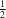 + *z*
C21⋯H12*D*	2.78	1 − *x*, 1 − *y*, 1 − *z*
H12*A*⋯H9*A*2	2.16	1 − *x*, − 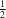 + *y*, 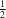 − *z*
H17⋯H26	2.18	1 + *x*, *y*, *z*
C4*A*⋯H9*A*1	2.75	1 − *x*, 1 − *y*, −*z*
S1*A*⋯H18	2.91	1 − *x*, 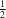 − *y*, − 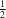 + *z*
H17⋯H13*A*	2.33	2 − *x*, 1 − *y*, 1 − *z*

Note: (*a*) The inter­atomic distances are calculated in *Crystal Explorer 17* (Turner *et al.*, 2017 ▸) whereby the *X*—H bond lengths are adjusted to their neutron values.

**Table 6:** Percentage contributions of inter­atomic contacts to the Hirshfeld surface for (I), the Sn1-mol­ecule in (II) and the Sn2-mol­ecule in (II)

Contact	Percentage contribution		
	(I)	Sn1-mol­ecule in (II)	Sn2-mol­ecule in (II)
H⋯H	62.2	59.9	64.9
C⋯H/H⋯C	28.4	24.3	20.1
S⋯H/H⋯S	8.6	14.4	13.6
N⋯H/H⋯ N	0.1	0.8	0.7
C⋯C	0.4	0.3	0.1
S⋯C/C⋯S	0.2	0.4	0.6
Sn⋯H/H⋯Sn	0.1	0.0	0.0

**Table 7:** Summary of inter­action energies (kJ mol^−1^) calculated for (I) and (II)

Contact	*R* (Å)	*E*_ele_	*E*_pol_	*E*_dis_	*E*_rep_	*E*_tot_
(I)^*a*^						
H13⋯C26^i^ +	8.06	−13.8	−5.6	−68.5	42.5	−45.0
C13—H13⋯*Cg*(C21–C26)^i^ +						
C23—H23⋯*Cg*(C31–C36)^i^ +						
H6⋯C12^i^ +						
H7*B*⋯S1^i^						
C16 ⋯H5*B* ^ii^	8.42	−21.8	−5.6	−52.2	29.1	−49.3
C33 ⋯H2*B* ^iii^ +	8.00	−21.2	−7.0	−59.2	29.5	−55.6
S1⋯H2*A* ^iii^						
C25⋯H4*B* ^iv^	9.91	−0.6	−0.8	−23.3	8.4	−15.2
H15⋯H24^v^	12.94	−2.6	−0.5	−12.5	9.1	−6.9
(II)^*b*^						
S1⋯C11*A* ^i^ +	8.68	−25.4	−8.6	−67.8	44.0	−57.0
S4⋯C12*A* ^i^ +						
C7—H7*B*⋯*Cg*(C21*A*–C26*A*)^i^ +						
C21⋯H12*D* ^i^						
C4*A*⋯H9*A*1^ii^	9.0	−28.8	−7.3	−69.0	49.0	−56.5
C7—H7*A*⋯*Cg*(C15*A*–C20*A*)^iii^ +	9.21	−19.6	−7.4	−61.3	33.7	−52.6
H17⋯H13*A* ^iii^						
H17⋯H26^iv^	9.62	−12.0	−5.0	−51.2	25.9	−40.6
C11⋯C23*A* ^v^	9.93	−10.2	−2.9	−44.1	23.5	−33.0
C11⋯H23*A* ^v^						
H12*A*⋯H9*A*2^vi^	10.81	−5.9	−2.4	−30.6	14.5	−23.4
S1*A*⋯H18^vii^	10.11	−5.2	−3.6	−34.3	18.9	−23.
C19⋯H11*B* ^viii^	12.51	−7.4	−2.6	−20.5	9.8	−19.8

Notes: (*a*) Symmetry operations for (I): (i) −1 + *x*, *y*, *z*; (ii) 1 − *x*, − *y*, 1 − *z*; (iii) 1 − *x*, 1 − *y*, 1 − *z*; (iv) *x* 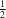 − *y*, −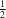 + *z*; (v) 1 − *x*, −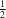 + *y*, 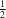 − *z*. (*b*) Symmetry operations for (II): (i) 1 − *x*, 1 − *y*, 1 − *z*; (ii) 1 − *x*, 1 − *y*, − *z*; (iii) 2 − *x*, 1 − *y*, 1 − *z*; (iv) 1 + *x*, *y*, *z*; (v) *x*, *y*, *z*; (vi) 1 − *x*, −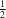 + *y*, 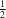 − *z*; (vii) 1 − *x*, 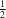 − *y*, −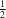 + *z*; (viii) 1 + *x*, 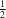 − *y*, 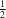 + *z*.

**Table 8:** Experimental details

	(I)	(II)
Crystal data		
Chemical formula	[Sn(C_6_H_5_)_3_(C_7_H_10_NS_2_)]	[Sn(C_6_H_5_)_2_(C_7_H_10_NS_2_)_2_]
*M*_r_	522.27	617.45
Crystal system, space group	Monoclinic, *P*2_1_/*c*	Monoclinic, *P*2_1_/*c*
Temperature (K)	100	100
*a*, *b*, *c* (Å)	8.0650 (1), 11.4490 (1), 25.8775 (2)	9.6160 (1), 30.4216 (2), 19.1928 (1)
β (°)	98.282 (1)	100.019 (1)
*V* (Å^3^)	2364.51 (4)	5528.93 (8)
*Z*	4	8
Radiation type	Cu *K*α	Cu *K*α
μ (mm^−1^)	10.32	10.30
Crystal size (mm)	0.13 × 0.10 × 0.04	0.19 × 0.14 × 0.07

Data collection		
Diffractometer	XtaLAB Synergy, Dualflex, AtlasS2	XtaLAB Synergy, Dualflex, AtlasS2
Absorption correction	Gaussian (*CrysAlis PRO*; Rigaku OD, 2018 ▸)	Gaussian (*CrysAlis PRO*; Rigaku OD, 2018 ▸)
*T*_min_, *T*_max_	0.807, 1.000	0.633, 1.000
No. of measured, independent and observed [*I* > 2σ(*I*)] reflections	55086, 4226, 4083	67735, 9876, 9370
*R*_int_	0.044	0.037
(sin θ/λ)_max_ (Å^−1^)	0.597	0.597

Refinement		
*R*[*F* ^2^ > 2σ(*F* ^2^)], *wR*(*F* ^2^), *S*	0.020, 0.053, 1.01	0.027, 0.068, 1.02
No. of reflections	4226	9876
No. of parameters	262	595
H-atom treatment	H-atom parameters constrained	H-atom parameters constrained
Δρ_max_, Δρ_min_ (e Å^−3^)	0.82, −0.50	1.23, −0.73

Computer programs: *CrysAlis PRO* (Rigaku OD, 2018 ▸), *SHELXS* (Sheldrick, 2015*a* ▸), *SHELXL2017/1* (Sheldrick, 2015*b* ▸), *ORTEP-3 for Windows* (Farrugia, 2012 ▸), *DIAMOND* (Brandenburg, 2006 ▸) and *publCIF* (Westrip, 2010 ▸).

## supplementary crystallographic information

### (N,N-Diallyldithiocarbamato-κ2S,S')\ triphenyltin(IV) (I) Crystal data

**Table:**

[Sn(C_6_H_5_)_3_(C_7_H_10_NS_2_)]	*F*(000) = 1056
*M**_r_* = 522.27	*D*_x_ = 1.467 Mg m^−^^3^
Monoclinic, *P*2_1_/*c*	Cu *K*α radiation, λ = 1.54184 Å
*a* = 8.0650 (1) Å	Cell parameters from 34049 reflections
*b* = 11.4490 (1) Å	θ = 3.4–76.3°
*c* = 25.8775 (2) Å	µ = 10.32 mm^−^^1^
β = 98.282 (1)°	*T* = 100 K
*V* = 2364.51 (4) Å^3^	Prism, colourless
*Z* = 4	0.13 × 0.10 × 0.04 mm

### (N,N-Diallyldithiocarbamato-κ2S,S')\ triphenyltin(IV) (I) Data collection

**Table:**

XtaLAB Synergy, Dualflex, AtlasS2 diffractometer	4226 independent reflections
Radiation source: micro-focus sealed X-ray tube, PhotonJet (Cu) X-ray Source	4083 reflections with *I* > 2σ(*I*)
Mirror monochromator	*R*_int_ = 0.044
Detector resolution: 5.2558 pixels mm^-1^	θ_max_ = 67.1°, θ_min_ = 3.5°
ω scans	*h* = −9→9
Absorption correction: gaussian (CrysAlis PRO; Rigaku OD, 2018)	*k* = −13→13
*T*_min_ = 0.807, *T*_max_ = 1.000	*l* = −26→30
55086 measured reflections	

### (N,N-Diallyldithiocarbamato-κ2S,S')\ triphenyltin(IV) (I) Refinement

**Table:**

Refinement on *F*^2^	Primary atom site location: structure-invariant direct methods
Least-squares matrix: full	Secondary atom site location: difference Fourier map
*R*[*F*^2^ > 2σ(*F*^2^)] = 0.020	Hydrogen site location: inferred from neighbouring sites
*wR*(*F*^2^) = 0.053	H-atom parameters constrained
*S* = 1.01	*w* = 1/[σ^2^(*F*_o_^2^) + (0.034*P*)^2^ + 1.0941*P*] where *P* = (*F*_o_^2^ + 2*F*_c_^2^)/3
4226 reflections	(Δ/σ)_max_ = 0.001
262 parameters	Δρ_max_ = 0.82 e Å^−^^3^
0 restraints	Δρ_min_ = −0.50 e Å^−^^3^

### (N,N-Diallyldithiocarbamato-κ2S,S')\ triphenyltin(IV) (I) Special details

**Table:**

Geometry. All esds (except the esd in the dihedral angle between two l.s. planes) are estimated using the full covariance matrix. The cell esds are taken into account individually in the estimation of esds in distances, angles and torsion angles; correlations between esds in cell parameters are only used when they are defined by crystal symmetry. An approximate (isotropic) treatment of cell esds is used for estimating esds involving l.s. planes.

### (N,N-Diallyldithiocarbamato-κ2S,S')\ triphenyltin(IV) (I) Fractional atomic coordinates and isotropic or equivalent isotropic displacement parameters (Å^2^)

**Table:**

	*x*	*y*	*z*	*U*_iso_*/*U*_eq_	
Sn	0.25851 (2)	0.27508 (2)	0.37027 (2)	0.01943 (6)	
S1	0.40753 (6)	0.34500 (4)	0.45492 (2)	0.02546 (11)	
S2	0.50865 (6)	0.11062 (4)	0.42287 (2)	0.02585 (11)	
N1	0.6650 (2)	0.22704 (13)	0.50510 (7)	0.0221 (3)	
C1	0.5424 (2)	0.22413 (16)	0.46477 (8)	0.0211 (4)	
C2	0.6883 (2)	0.32426 (18)	0.54246 (8)	0.0267 (4)	
H2A	0.646687	0.397215	0.524565	0.032*	
H2B	0.809419	0.334224	0.554922	0.032*	
C3	0.5978 (3)	0.3042 (2)	0.58847 (8)	0.0309 (5)	
H3	0.612847	0.360684	0.615638	0.037*	
C4	0.4992 (3)	0.2149 (2)	0.59435 (9)	0.0319 (5)	
H4A	0.480682	0.156449	0.568121	0.038*	
H4B	0.446654	0.209057	0.624828	0.038*	
C5	0.7846 (2)	0.12945 (18)	0.51605 (8)	0.0249 (4)	
H5A	0.732148	0.056406	0.501157	0.030*	
H5B	0.812801	0.118925	0.554284	0.030*	
C6	0.9410 (3)	0.1527 (2)	0.49311 (9)	0.0315 (4)	
H6	0.931935	0.161093	0.456289	0.038*	
C7	1.0904 (3)	0.1621 (2)	0.52078 (11)	0.0427 (6)	
H7A	1.103510	0.154114	0.557684	0.051*	
H7B	1.185305	0.176968	0.503870	0.051*	
C11	0.0883 (2)	0.13134 (16)	0.35523 (7)	0.0204 (4)	
C12	−0.0823 (2)	0.15586 (18)	0.34399 (7)	0.0257 (4)	
H12	−0.120299	0.233677	0.347295	0.031*	
C13	−0.1979 (2)	0.06837 (19)	0.32802 (8)	0.0278 (4)	
H13	−0.313765	0.086573	0.320597	0.033*	
C14	−0.1442 (3)	−0.04484 (19)	0.32295 (8)	0.0279 (4)	
H14	−0.222732	−0.104752	0.311737	0.033*	
C15	0.0259 (3)	−0.07077 (18)	0.33438 (9)	0.0308 (4)	
H15	0.063245	−0.148801	0.331218	0.037*	
C16	0.1410 (2)	0.01651 (17)	0.35033 (8)	0.0254 (4)	
H16	0.256747	−0.002093	0.357990	0.030*	
C21	0.4161 (2)	0.28918 (16)	0.31120 (7)	0.0213 (4)	
C22	0.5403 (2)	0.37446 (18)	0.31258 (8)	0.0269 (4)	
H22	0.554696	0.429581	0.340305	0.032*	
C23	0.6428 (3)	0.37983 (19)	0.27407 (9)	0.0312 (5)	
H23	0.726432	0.438594	0.275407	0.037*	
C24	0.6234 (3)	0.2996 (2)	0.23361 (9)	0.0313 (5)	
H24	0.693634	0.303209	0.207178	0.038*	
C25	0.5010 (3)	0.21372 (18)	0.23174 (8)	0.0280 (4)	
H25	0.487954	0.158250	0.204148	0.034*	
C26	0.3979 (2)	0.20907 (17)	0.27018 (8)	0.0223 (4)	
H26	0.313726	0.150577	0.268562	0.027*	
C31	0.0978 (2)	0.42764 (17)	0.36369 (8)	0.0242 (4)	
C32	−0.0065 (3)	0.45310 (18)	0.40077 (8)	0.0293 (4)	
H32	−0.007314	0.402694	0.429894	0.035*	
C33	−0.1095 (3)	0.5512 (2)	0.39575 (10)	0.0357 (5)	
H33	−0.180213	0.567475	0.421210	0.043*	
C34	−0.1081 (3)	0.62513 (19)	0.35328 (10)	0.0374 (5)	
H34	−0.177793	0.692406	0.349818	0.045*	
C35	−0.0067 (3)	0.60157 (19)	0.31626 (9)	0.0350 (5)	
H35	−0.006485	0.652566	0.287313	0.042*	
C36	0.0959 (2)	0.50318 (18)	0.32102 (8)	0.0273 (4)	
H36	0.165193	0.487164	0.295126	0.033*	

### (N,N-Diallyldithiocarbamato-κ2S,S')\ triphenyltin(IV) (I) Atomic displacement parameters (Å^2^)

**Table:**

	*U*^11^	*U*^22^	*U*^33^	*U*^12^	*U*^13^	*U*^23^
Sn	0.02063 (8)	0.01640 (8)	0.02001 (8)	0.00251 (4)	−0.00135 (5)	−0.00007 (4)
S1	0.0271 (2)	0.0215 (2)	0.0253 (2)	0.00683 (18)	−0.00475 (18)	−0.00472 (17)
S2	0.0301 (2)	0.0205 (2)	0.0254 (2)	0.00408 (18)	−0.00133 (18)	−0.00396 (18)
N1	0.0206 (8)	0.0200 (9)	0.0246 (8)	0.0016 (6)	−0.0007 (7)	0.0000 (6)
C1	0.0186 (9)	0.0212 (10)	0.0232 (9)	0.0002 (7)	0.0025 (7)	0.0024 (7)
C2	0.0249 (10)	0.0223 (10)	0.0303 (10)	−0.0022 (8)	−0.0051 (8)	−0.0028 (8)
C3	0.0314 (11)	0.0333 (11)	0.0255 (10)	0.0014 (9)	−0.0043 (8)	−0.0109 (9)
C4	0.0289 (11)	0.0404 (13)	0.0257 (10)	0.0021 (9)	0.0015 (8)	−0.0050 (8)
C5	0.0215 (9)	0.0240 (10)	0.0282 (10)	0.0029 (8)	0.0005 (7)	0.0037 (8)
C6	0.0285 (10)	0.0312 (11)	0.0356 (11)	0.0057 (9)	0.0074 (9)	0.0036 (9)
C7	0.0255 (11)	0.0398 (14)	0.0632 (16)	0.0002 (10)	0.0083 (10)	0.0010 (12)
C11	0.0245 (9)	0.0192 (9)	0.0173 (8)	0.0018 (7)	0.0029 (7)	0.0011 (7)
C12	0.0245 (9)	0.0241 (10)	0.0270 (9)	0.0061 (8)	−0.0013 (8)	−0.0026 (8)
C13	0.0212 (9)	0.0328 (11)	0.0279 (10)	0.0015 (8)	−0.0011 (7)	0.0008 (8)
C14	0.0278 (10)	0.0268 (11)	0.0296 (10)	−0.0080 (8)	0.0059 (8)	−0.0019 (8)
C15	0.0298 (10)	0.0187 (10)	0.0453 (12)	−0.0004 (8)	0.0107 (9)	−0.0025 (9)
C16	0.0233 (9)	0.0190 (10)	0.0347 (10)	0.0017 (8)	0.0069 (8)	−0.0005 (8)
C21	0.0203 (9)	0.0204 (9)	0.0215 (9)	0.0019 (7)	−0.0024 (7)	0.0036 (7)
C22	0.0260 (10)	0.0203 (10)	0.0320 (10)	−0.0032 (8)	−0.0037 (8)	0.0013 (8)
C23	0.0235 (10)	0.0288 (11)	0.0396 (11)	−0.0078 (8)	−0.0012 (8)	0.0090 (9)
C24	0.0230 (10)	0.0387 (12)	0.0325 (11)	−0.0006 (9)	0.0051 (8)	0.0097 (9)
C25	0.0276 (10)	0.0297 (11)	0.0258 (10)	−0.0019 (8)	0.0006 (8)	0.0010 (8)
C26	0.0206 (9)	0.0224 (9)	0.0227 (9)	−0.0032 (7)	−0.0004 (7)	0.0022 (7)
C31	0.0226 (9)	0.0154 (9)	0.0318 (10)	0.0013 (7)	−0.0053 (8)	−0.0033 (7)
C32	0.0284 (10)	0.0207 (10)	0.0366 (11)	0.0016 (8)	−0.0022 (8)	−0.0044 (8)
C33	0.0245 (10)	0.0270 (11)	0.0534 (13)	0.0010 (9)	−0.0018 (9)	−0.0144 (10)
C34	0.0263 (10)	0.0179 (10)	0.0621 (15)	0.0029 (8)	−0.0139 (10)	−0.0059 (10)
C35	0.0306 (11)	0.0205 (10)	0.0481 (13)	−0.0019 (9)	−0.0140 (10)	0.0033 (9)
C36	0.0247 (9)	0.0201 (10)	0.0331 (10)	−0.0016 (8)	−0.0099 (8)	−0.0001 (8)

### (N,N-Diallyldithiocarbamato-κ2S,S')\ triphenyltin(IV) (I) Geometric parameters (Å, º)

**Table:**

Sn—C21	2.130 (2)	C13—H13	0.9500
Sn—C11	2.1427 (19)	C14—C15	1.393 (3)
Sn—C31	2.1673 (19)	C14—H14	0.9500
Sn—S1	2.4749 (4)	C15—C16	1.386 (3)
Sn—S2	2.9456 (5)	C15—H15	0.9500
S1—C1	1.7559 (19)	C16—H16	0.9500
S2—C1	1.6894 (19)	C21—C26	1.394 (3)
N1—C1	1.330 (3)	C21—C22	1.395 (3)
N1—C2	1.468 (3)	C22—C23	1.384 (3)
N1—C5	1.477 (2)	C22—H22	0.9500
C2—C3	1.502 (3)	C23—C24	1.385 (3)
C2—H2A	0.9900	C23—H23	0.9500
C2—H2B	0.9900	C24—C25	1.388 (3)
C3—C4	1.317 (3)	C24—H24	0.9500
C3—H3	0.9500	C25—C26	1.387 (3)
C4—H4A	0.9500	C25—H25	0.9500
C4—H4B	0.9500	C26—H26	0.9500
C5—C6	1.493 (3)	C31—C32	1.395 (3)
C5—H5A	0.9900	C31—C36	1.401 (3)
C5—H5B	0.9900	C32—C33	1.392 (3)
C6—C7	1.315 (3)	C32—H32	0.9500
C6—H6	0.9500	C33—C34	1.388 (4)
C7—H7A	0.9500	C33—H33	0.9500
C7—H7B	0.9500	C34—C35	1.373 (4)
C11—C12	1.393 (3)	C34—H34	0.9500
C11—C16	1.393 (3)	C35—C36	1.393 (3)
C12—C13	1.390 (3)	C35—H35	0.9500
C12—H12	0.9500	C36—H36	0.9500
C13—C14	1.379 (3)		
			
C21—Sn—C11	111.15 (7)	C14—C13—C12	119.99 (18)
C21—Sn—C31	107.13 (7)	C14—C13—H13	120.0
C11—Sn—C31	104.14 (7)	C12—C13—H13	120.0
C21—Sn—S1	110.28 (5)	C13—C14—C15	119.53 (19)
C11—Sn—S1	128.76 (5)	C13—C14—H14	120.2
C31—Sn—S1	91.01 (5)	C15—C14—H14	120.2
C21—Sn—S2	86.57 (5)	C16—C15—C14	120.43 (19)
C11—Sn—S2	88.36 (5)	C16—C15—H15	119.8
C31—Sn—S2	156.01 (5)	C14—C15—H15	119.8
S1—Sn—S2	65.470 (14)	C15—C16—C11	120.48 (18)
C1—S1—Sn	94.89 (7)	C15—C16—H16	119.8
C1—S2—Sn	80.86 (6)	C11—C16—H16	119.8
C1—N1—C2	123.08 (16)	C26—C21—C22	118.46 (19)
C1—N1—C5	121.51 (16)	C26—C21—Sn	119.19 (14)
C2—N1—C5	115.39 (15)	C22—C21—Sn	122.33 (15)
N1—C1—S2	123.75 (14)	C23—C22—C21	120.87 (19)
N1—C1—S1	117.96 (14)	C23—C22—H22	119.6
S2—C1—S1	118.29 (11)	C21—C22—H22	119.6
N1—C2—C3	112.04 (17)	C22—C23—C24	120.03 (19)
N1—C2—H2A	109.2	C22—C23—H23	120.0
C3—C2—H2A	109.2	C24—C23—H23	120.0
N1—C2—H2B	109.2	C23—C24—C25	119.9 (2)
C3—C2—H2B	109.2	C23—C24—H24	120.1
H2A—C2—H2B	107.9	C25—C24—H24	120.1
C4—C3—C2	125.52 (19)	C26—C25—C24	119.9 (2)
C4—C3—H3	117.2	C26—C25—H25	120.0
C2—C3—H3	117.2	C24—C25—H25	120.0
C3—C4—H4A	120.0	C25—C26—C21	120.81 (18)
C3—C4—H4B	120.0	C25—C26—H26	119.6
H4A—C4—H4B	120.0	C21—C26—H26	119.6
N1—C5—C6	110.80 (17)	C32—C31—C36	118.31 (19)
N1—C5—H5A	109.5	C32—C31—Sn	121.84 (15)
C6—C5—H5A	109.5	C36—C31—Sn	119.85 (15)
N1—C5—H5B	109.5	C31—C32—C33	121.0 (2)
C6—C5—H5B	109.5	C31—C32—H32	119.5
H5A—C5—H5B	108.1	C33—C32—H32	119.5
C7—C6—C5	124.0 (2)	C34—C33—C32	119.6 (2)
C7—C6—H6	118.0	C34—C33—H33	120.2
C5—C6—H6	118.0	C32—C33—H33	120.2
C6—C7—H7A	120.0	C35—C34—C33	120.4 (2)
C6—C7—H7B	120.0	C35—C34—H34	119.8
H7A—C7—H7B	120.0	C33—C34—H34	119.8
C12—C11—C16	118.46 (18)	C34—C35—C36	120.2 (2)
C12—C11—Sn	118.09 (14)	C34—C35—H35	119.9
C16—C11—Sn	123.03 (14)	C36—C35—H35	119.9
C13—C12—C11	121.09 (19)	C35—C36—C31	120.5 (2)
C13—C12—H12	119.5	C35—C36—H36	119.7
C11—C12—H12	119.5	C31—C36—H36	119.7
			
C2—N1—C1—S2	−177.36 (15)	C14—C15—C16—C11	0.1 (3)
C5—N1—C1—S2	0.8 (3)	C12—C11—C16—C15	0.3 (3)
C2—N1—C1—S1	2.3 (3)	Sn—C11—C16—C15	−172.13 (15)
C5—N1—C1—S1	−179.62 (14)	C26—C21—C22—C23	0.2 (3)
Sn—S2—C1—N1	−174.18 (18)	Sn—C21—C22—C23	178.75 (15)
Sn—S2—C1—S1	6.21 (10)	C21—C22—C23—C24	−0.3 (3)
Sn—S1—C1—N1	173.04 (15)	C22—C23—C24—C25	0.0 (3)
Sn—S1—C1—S2	−7.33 (12)	C23—C24—C25—C26	0.4 (3)
C1—N1—C2—C3	91.6 (2)	C24—C25—C26—C21	−0.5 (3)
C5—N1—C2—C3	−86.6 (2)	C22—C21—C26—C25	0.2 (3)
N1—C2—C3—C4	−4.2 (3)	Sn—C21—C26—C25	−178.38 (15)
C1—N1—C5—C6	95.9 (2)	C36—C31—C32—C33	−0.3 (3)
C2—N1—C5—C6	−85.9 (2)	Sn—C31—C32—C33	−179.70 (14)
N1—C5—C6—C7	118.2 (2)	C31—C32—C33—C34	−0.2 (3)
C16—C11—C12—C13	−0.3 (3)	C32—C33—C34—C35	0.3 (3)
Sn—C11—C12—C13	172.55 (15)	C33—C34—C35—C36	0.0 (3)
C11—C12—C13—C14	−0.2 (3)	C34—C35—C36—C31	−0.4 (3)
C12—C13—C14—C15	0.6 (3)	C32—C31—C36—C35	0.6 (3)
C13—C14—C15—C16	−0.5 (3)	Sn—C31—C36—C35	−179.98 (14)

### (N,N-Diallyldithiocarbamato-κ2S,S')\ triphenyltin(IV) (I) Hydrogen-bond geometry (Å, º)

*Cg*1 and *Cg*2 are the centroids of the (C21–C26) and (C31–C36) rings, respectively.

**Table:**

*D*—H···*A*	*D*—H	H···*A*	*D*···*A*	*D*—H···*A*
C13—H13···*Cg*1^i^	0.95	2.92	3.605 (2)	130
C23—H23···*Cg*2^ii^	0.95	2.99	3.720 (3)	134

Symmetry codes: (i) *x*−1, *y*, *z*; (ii) *x*+1, *y*, *z*.

### Bis(N,N-diallyldithiocarbamato-κ2S,S')diphenyltin(IV) (II) Crystal data

**Table:**

[Sn(C_6_H_5_)_2_(C_7_H_10_NS_2_)_2_]	*F*(000) = 2512
*M**_r_* = 617.45	*D*_x_ = 1.484 Mg m^−^^3^
Monoclinic, *P*2_1_/*c*	Cu *K*α radiation, λ = 1.54184 Å
*a* = 9.6160 (1) Å	Cell parameters from 40535 reflections
*b* = 30.4216 (2) Å	θ = 2.9–76.3°
*c* = 19.1928 (1) Å	µ = 10.30 mm^−^^1^
β = 100.019 (1)°	*T* = 100 K
*V* = 5528.93 (8) Å^3^	Prism, colourless
*Z* = 8	0.19 × 0.14 × 0.07 mm

### Bis(N,N-diallyldithiocarbamato-κ2S,S')diphenyltin(IV) (II) Data collection

**Table:**

XtaLAB Synergy, Dualflex, AtlasS2 diffractometer	9876 independent reflections
Radiation source: micro-focus sealed X-ray tube, PhotonJet (Cu) X-ray Source	9370 reflections with *I* > 2σ(*I*)
Mirror monochromator	*R*_int_ = 0.037
Detector resolution: 5.2558 pixels mm^-1^	θ_max_ = 67.1°, θ_min_ = 3.7°
ω scans	*h* = −11→11
Absorption correction: gaussian (CrysAlisPro; Rigaku OD, 2018)	*k* = −36→25
*T*_min_ = 0.633, *T*_max_ = 1.000	*l* = −22→22
67735 measured reflections	

### Bis(N,N-diallyldithiocarbamato-κ2S,S')diphenyltin(IV) (II) Refinement

**Table:**

Refinement on *F*^2^	Primary atom site location: structure-invariant direct methods
Least-squares matrix: full	Secondary atom site location: difference Fourier map
*R*[*F*^2^ > 2σ(*F*^2^)] = 0.027	Hydrogen site location: inferred from neighbouring sites
*wR*(*F*^2^) = 0.068	H-atom parameters constrained
*S* = 1.01	*w* = 1/[σ^2^(*F*_o_^2^) + (0.0362*P*)^2^ + 7.0312*P*] where *P* = (*F*_o_^2^ + 2*F*_c_^2^)/3
9876 reflections	(Δ/σ)_max_ = 0.003
595 parameters	Δρ_max_ = 1.23 e Å^−^^3^
0 restraints	Δρ_min_ = −0.73 e Å^−^^3^

### Bis(N,N-diallyldithiocarbamato-κ2S,S')diphenyltin(IV) (II) Special details

**Table:**

Geometry. All esds (except the esd in the dihedral angle between two l.s. planes) are estimated using the full covariance matrix. The cell esds are taken into account individually in the estimation of esds in distances, angles and torsion angles; correlations between esds in cell parameters are only used when they are defined by crystal symmetry. An approximate (isotropic) treatment of cell esds is used for estimating esds involving l.s. planes.

### Bis(N,N-diallyldithiocarbamato-κ2S,S')diphenyltin(IV) (II) Fractional atomic coordinates and isotropic or equivalent isotropic displacement parameters (Å^2^)

**Table:**

	*x*	*y*	*z*	*U*_iso_*/*U*_eq_	
Sn1	0.96840 (2)	0.26758 (2)	0.60783 (2)	0.01863 (5)	
S1	1.05093 (7)	0.34237 (2)	0.65910 (3)	0.02202 (13)	
S2	1.05034 (7)	0.32188 (2)	0.50854 (3)	0.02481 (13)	
S3	0.82312 (7)	0.21606 (2)	0.51668 (4)	0.02761 (14)	
S4	0.71629 (7)	0.30174 (2)	0.55382 (3)	0.02345 (13)	
N1	1.1264 (2)	0.39971 (7)	0.56894 (11)	0.0203 (4)	
N2	0.5656 (2)	0.24617 (7)	0.46333 (12)	0.0253 (5)	
C1	1.0824 (2)	0.35895 (8)	0.57638 (13)	0.0189 (5)	
C2	1.1517 (3)	0.41701 (9)	0.50102 (14)	0.0251 (6)	
H2A	1.121986	0.448186	0.496767	0.030*	
H2B	1.093159	0.400473	0.462164	0.030*	
C3	1.3039 (3)	0.41377 (9)	0.49308 (14)	0.0286 (6)	
H3	1.331520	0.429629	0.455081	0.034*	
C4	1.4027 (3)	0.39104 (10)	0.53379 (16)	0.0309 (6)	
H4A	1.380189	0.374657	0.572463	0.037*	
H4B	1.496585	0.391033	0.524485	0.037*	
C5	1.1518 (3)	0.43140 (8)	0.62834 (14)	0.0225 (5)	
H5A	1.229774	0.451427	0.621918	0.027*	
H5B	1.180874	0.415255	0.673303	0.027*	
C6	1.0221 (3)	0.45784 (9)	0.63244 (13)	0.0246 (5)	
H6	0.938442	0.442486	0.637625	0.030*	
C7	1.0169 (3)	0.50095 (10)	0.62931 (15)	0.0312 (6)	
H7A	1.098766	0.517238	0.624141	0.037*	
H7B	0.931250	0.515847	0.632218	0.037*	
C8	0.6860 (3)	0.25402 (8)	0.50603 (13)	0.0211 (5)	
C9	0.4473 (3)	0.27826 (9)	0.45194 (15)	0.0270 (6)	
H9A	0.459611	0.299926	0.491021	0.032*	
H9B	0.356671	0.262729	0.451561	0.032*	
C10	0.4444 (3)	0.30145 (11)	0.38295 (16)	0.0357 (7)	
H10	0.513805	0.323315	0.380204	0.043*	
C11	0.3516 (4)	0.29318 (13)	0.32636 (18)	0.0485 (9)	
H11A	0.281054	0.271475	0.327679	0.058*	
H11B	0.354463	0.308876	0.283842	0.058*	
C12	0.5396 (3)	0.20533 (10)	0.42143 (16)	0.0341 (7)	
H12A	0.630727	0.190610	0.419544	0.041*	
H12B	0.494638	0.212628	0.372403	0.041*	
C13	0.4460 (4)	0.17484 (10)	0.45334 (18)	0.0415 (8)	
H13	0.483016	0.161495	0.497427	0.050*	
C14	0.3162 (4)	0.16547 (13)	0.4240 (3)	0.0601 (11)	
H14A	0.276367	0.178312	0.379956	0.072*	
H14B	0.261771	0.145828	0.446853	0.072*	
C15	1.1604 (3)	0.22965 (8)	0.61670 (13)	0.0210 (5)	
C16	1.2902 (3)	0.24774 (9)	0.60881 (14)	0.0247 (5)	
H16	1.295812	0.277867	0.596567	0.030*	
C17	1.4111 (3)	0.22188 (9)	0.61878 (16)	0.0289 (6)	
H17	1.499314	0.234539	0.614001	0.035*	
C18	1.4039 (3)	0.17786 (9)	0.63562 (14)	0.0273 (6)	
H18	1.486760	0.160247	0.641839	0.033*	
C19	1.2754 (3)	0.15942 (9)	0.64342 (14)	0.0256 (6)	
H19	1.270001	0.129177	0.654966	0.031*	
C20	1.1552 (3)	0.18534 (9)	0.63427 (13)	0.0230 (5)	
H20	1.067687	0.172668	0.640090	0.028*	
C21	0.8975 (3)	0.24718 (8)	0.70441 (13)	0.0203 (5)	
C22	0.9742 (3)	0.26067 (9)	0.76923 (14)	0.0265 (6)	
H22	1.055704	0.278445	0.770037	0.032*	
C23	0.9342 (3)	0.24871 (10)	0.83264 (15)	0.0323 (6)	
H23	0.988359	0.258258	0.876175	0.039*	
C24	0.8158 (3)	0.22295 (10)	0.83270 (16)	0.0317 (6)	
H24	0.787943	0.214921	0.876082	0.038*	
C25	0.7383 (3)	0.20897 (10)	0.76898 (16)	0.0327 (6)	
H25	0.656701	0.191282	0.768520	0.039*	
C26	0.7797 (3)	0.22080 (9)	0.70546 (15)	0.0266 (6)	
H26	0.726533	0.210653	0.662062	0.032*	
Sn2	0.42934 (2)	0.47935 (2)	0.26224 (2)	0.01967 (5)	
S1A	0.50653 (7)	0.43729 (2)	0.15999 (3)	0.02630 (14)	
S2A	0.65505 (7)	0.51843 (2)	0.21454 (3)	0.02630 (14)	
S3A	0.39019 (7)	0.55322 (2)	0.32085 (3)	0.02589 (14)	
S4A	0.29651 (7)	0.53675 (2)	0.16787 (3)	0.02586 (14)	
N1A	0.6984 (2)	0.47703 (7)	0.09775 (12)	0.0264 (5)	
N2A	0.2774 (2)	0.61450 (7)	0.22935 (11)	0.0229 (4)	
C1A	0.6292 (3)	0.47819 (8)	0.15187 (14)	0.0234 (5)	
C2A	0.6696 (3)	0.44435 (10)	0.03990 (15)	0.0328 (6)	
H2A1	0.675651	0.458786	−0.005706	0.039*	
H2A2	0.572710	0.432678	0.037076	0.039*	
C3A	0.7733 (4)	0.40729 (10)	0.05215 (16)	0.0375 (7)	
H3A	0.763123	0.386079	0.087245	0.045*	
C4A	0.8781 (4)	0.40263 (11)	0.01660 (17)	0.0400 (7)	
H4A1	0.890527	0.423401	−0.018758	0.048*	
H4A2	0.941186	0.378490	0.026373	0.048*	
C5A	0.8112 (3)	0.50881 (10)	0.09071 (15)	0.0280 (6)	
H5A1	0.888230	0.493556	0.072253	0.034*	
H5A2	0.850815	0.520988	0.137826	0.034*	
C6A	0.7561 (4)	0.54519 (12)	0.04217 (19)	0.0446 (8)	
H6A	0.688042	0.564544	0.055802	0.053*	
C7A	0.7971 (5)	0.55184 (17)	−0.0189 (2)	0.0720 (15)	
H7A1	0.865062	0.532902	−0.033670	0.086*	
H7A2	0.758874	0.575581	−0.048313	0.086*	
C8A	0.3158 (3)	0.57268 (8)	0.23782 (13)	0.0221 (5)	
C9A	0.2113 (3)	0.63244 (9)	0.16070 (14)	0.0254 (6)	
H9A1	0.250124	0.617093	0.122830	0.030*	
H9A2	0.236138	0.663945	0.158775	0.030*	
C10A	0.0529 (3)	0.62794 (9)	0.14665 (14)	0.0270 (6)	
H10A	0.003035	0.643092	0.106705	0.032*	
C11A	−0.0226 (3)	0.60500 (9)	0.18449 (15)	0.0296 (6)	
H11C	0.022624	0.589294	0.224927	0.036*	
H11D	−0.122521	0.604126	0.171420	0.036*	
C12A	0.3060 (3)	0.64644 (9)	0.28729 (15)	0.0262 (6)	
H12C	0.306030	0.631114	0.332786	0.031*	
H12D	0.229511	0.668611	0.281445	0.031*	
C13A	0.4451 (3)	0.66935 (9)	0.28974 (15)	0.0304 (6)	
H13A	0.461271	0.694727	0.318873	0.036*	
C14A	0.5464 (3)	0.65786 (11)	0.25573 (16)	0.0345 (7)	
H14C	0.535363	0.632722	0.225908	0.041*	
H14D	0.630812	0.674669	0.260903	0.041*	
C15A	0.5755 (3)	0.45369 (8)	0.35224 (13)	0.0199 (5)	
C16A	0.6517 (3)	0.41536 (9)	0.34627 (15)	0.0262 (6)	
H16A	0.641902	0.400827	0.301871	0.031*	
C17A	0.7420 (3)	0.39798 (9)	0.40436 (16)	0.0281 (6)	
H17A	0.792462	0.371612	0.399584	0.034*	
C18A	0.7580 (3)	0.41916 (9)	0.46894 (15)	0.0267 (6)	
H18A	0.819483	0.407280	0.508595	0.032*	
C19A	0.6849 (3)	0.45768 (9)	0.47610 (14)	0.0263 (6)	
H19A	0.696848	0.472399	0.520395	0.032*	
C20A	0.5935 (3)	0.47474 (9)	0.41787 (14)	0.0222 (5)	
H20A	0.542863	0.501025	0.422959	0.027*	
C21A	0.2380 (3)	0.44192 (9)	0.26420 (13)	0.0219 (5)	
C22A	0.2467 (3)	0.40539 (9)	0.30792 (14)	0.0263 (6)	
H22A	0.335183	0.397555	0.335339	0.032*	
C23A	0.1275 (3)	0.37983 (10)	0.31248 (16)	0.0322 (6)	
H23A	0.135091	0.355207	0.343351	0.039*	
C24A	−0.0010 (3)	0.39055 (10)	0.27192 (16)	0.0330 (6)	
H24A	−0.082123	0.373216	0.274518	0.040*	
C25A	−0.0111 (3)	0.42636 (11)	0.22781 (17)	0.0387 (7)	
H25A	−0.099646	0.433692	0.199882	0.046*	
C26A	0.1072 (3)	0.45212 (10)	0.22356 (16)	0.0333 (6)	
H26A	0.098571	0.476793	0.192762	0.040*	

### Bis(N,N-diallyldithiocarbamato-κ2S,S')diphenyltin(IV) (II) Atomic displacement parameters (Å^2^)

**Table:**

	*U*^11^	*U*^22^	*U*^33^	*U*^12^	*U*^13^	*U*^23^
Sn1	0.01673 (9)	0.01771 (9)	0.02066 (9)	0.00008 (6)	0.00108 (6)	0.00380 (6)
S1	0.0246 (3)	0.0185 (3)	0.0228 (3)	−0.0030 (2)	0.0037 (2)	0.0032 (2)
S2	0.0267 (3)	0.0227 (3)	0.0238 (3)	0.0006 (2)	0.0011 (2)	−0.0032 (2)
S3	0.0280 (3)	0.0230 (3)	0.0299 (3)	0.0049 (3)	−0.0003 (3)	−0.0037 (3)
S4	0.0229 (3)	0.0177 (3)	0.0279 (3)	−0.0001 (2)	−0.0008 (2)	0.0003 (2)
N1	0.0208 (10)	0.0202 (10)	0.0198 (10)	−0.0006 (8)	0.0031 (8)	0.0034 (8)
N2	0.0270 (12)	0.0218 (11)	0.0267 (11)	0.0017 (9)	0.0031 (9)	−0.0053 (9)
C1	0.0168 (11)	0.0178 (12)	0.0212 (12)	0.0021 (9)	0.0004 (9)	0.0021 (10)
C2	0.0292 (14)	0.0255 (13)	0.0203 (13)	−0.0022 (11)	0.0035 (11)	0.0061 (10)
C3	0.0339 (15)	0.0307 (15)	0.0231 (13)	−0.0093 (12)	0.0099 (11)	−0.0019 (11)
C4	0.0255 (14)	0.0323 (15)	0.0362 (15)	−0.0045 (12)	0.0093 (12)	−0.0052 (12)
C5	0.0251 (13)	0.0183 (12)	0.0236 (13)	−0.0054 (10)	0.0026 (10)	−0.0003 (10)
C6	0.0282 (14)	0.0231 (13)	0.0218 (13)	−0.0027 (11)	0.0026 (10)	−0.0005 (10)
C7	0.0343 (15)	0.0277 (15)	0.0315 (15)	0.0037 (12)	0.0056 (12)	0.0024 (12)
C8	0.0230 (13)	0.0207 (12)	0.0198 (12)	0.0003 (10)	0.0046 (10)	0.0007 (10)
C9	0.0195 (13)	0.0303 (14)	0.0299 (14)	0.0020 (11)	0.0010 (11)	−0.0008 (12)
C10	0.0301 (15)	0.0400 (17)	0.0379 (17)	0.0053 (13)	0.0085 (13)	0.0092 (13)
C11	0.052 (2)	0.057 (2)	0.0348 (17)	0.0146 (18)	0.0035 (15)	0.0080 (16)
C12	0.0331 (15)	0.0317 (15)	0.0350 (16)	−0.0014 (13)	−0.0007 (12)	−0.0153 (13)
C13	0.057 (2)	0.0245 (15)	0.0402 (17)	−0.0038 (14)	0.0013 (15)	−0.0074 (13)
C14	0.055 (2)	0.042 (2)	0.080 (3)	−0.0198 (18)	0.004 (2)	−0.0071 (19)
C15	0.0203 (12)	0.0222 (13)	0.0199 (12)	0.0026 (10)	0.0023 (10)	−0.0008 (10)
C16	0.0245 (13)	0.0198 (13)	0.0304 (14)	0.0001 (11)	0.0061 (11)	0.0016 (11)
C17	0.0225 (13)	0.0259 (14)	0.0398 (16)	0.0013 (11)	0.0091 (12)	−0.0009 (12)
C18	0.0282 (14)	0.0237 (13)	0.0299 (14)	0.0080 (11)	0.0047 (11)	−0.0004 (11)
C19	0.0348 (15)	0.0172 (12)	0.0244 (13)	0.0003 (11)	0.0035 (11)	0.0013 (10)
C20	0.0223 (12)	0.0228 (13)	0.0231 (13)	−0.0025 (10)	0.0018 (10)	0.0013 (10)
C21	0.0212 (12)	0.0168 (12)	0.0234 (12)	0.0043 (10)	0.0057 (10)	0.0053 (10)
C22	0.0278 (14)	0.0239 (13)	0.0269 (14)	−0.0019 (11)	0.0024 (11)	0.0047 (11)
C23	0.0414 (17)	0.0289 (15)	0.0261 (14)	0.0008 (13)	0.0043 (12)	0.0041 (12)
C24	0.0372 (16)	0.0282 (14)	0.0332 (15)	0.0069 (12)	0.0155 (13)	0.0084 (12)
C25	0.0284 (14)	0.0314 (15)	0.0400 (17)	−0.0041 (12)	0.0107 (12)	0.0059 (13)
C26	0.0227 (13)	0.0284 (14)	0.0288 (14)	−0.0011 (11)	0.0049 (11)	0.0037 (11)
Sn2	0.02228 (9)	0.01617 (9)	0.01949 (9)	0.00243 (6)	0.00069 (6)	−0.00094 (6)
S1A	0.0294 (3)	0.0214 (3)	0.0284 (3)	−0.0055 (3)	0.0061 (3)	−0.0041 (2)
S2A	0.0315 (3)	0.0225 (3)	0.0245 (3)	−0.0041 (3)	0.0036 (3)	−0.0031 (2)
S3A	0.0329 (3)	0.0218 (3)	0.0211 (3)	0.0039 (3)	−0.0005 (3)	−0.0011 (2)
S4A	0.0346 (3)	0.0196 (3)	0.0214 (3)	0.0040 (3)	−0.0007 (3)	−0.0014 (2)
N1A	0.0283 (12)	0.0245 (12)	0.0254 (12)	−0.0052 (9)	0.0021 (9)	−0.0036 (9)
N2A	0.0248 (11)	0.0191 (11)	0.0240 (11)	0.0014 (9)	0.0024 (9)	−0.0015 (9)
C1A	0.0246 (13)	0.0213 (13)	0.0233 (13)	−0.0004 (10)	0.0012 (10)	0.0016 (10)
C2A	0.0380 (16)	0.0343 (16)	0.0260 (14)	−0.0104 (13)	0.0057 (12)	−0.0071 (12)
C3A	0.055 (2)	0.0259 (15)	0.0326 (16)	−0.0069 (14)	0.0099 (14)	−0.0063 (12)
C4A	0.051 (2)	0.0301 (16)	0.0383 (17)	−0.0024 (14)	0.0071 (15)	−0.0092 (13)
C5A	0.0270 (14)	0.0298 (14)	0.0267 (14)	−0.0035 (12)	0.0035 (11)	−0.0005 (11)
C6A	0.0364 (17)	0.0426 (19)	0.050 (2)	−0.0105 (15)	−0.0057 (15)	0.0169 (16)
C7A	0.070 (3)	0.093 (3)	0.043 (2)	−0.045 (3)	−0.0174 (19)	0.031 (2)
C8A	0.0227 (12)	0.0203 (13)	0.0228 (12)	0.0001 (10)	0.0028 (10)	−0.0008 (10)
C9A	0.0285 (14)	0.0210 (13)	0.0267 (13)	0.0034 (11)	0.0049 (11)	0.0049 (11)
C10A	0.0296 (14)	0.0238 (13)	0.0260 (13)	0.0057 (11)	0.0004 (11)	−0.0008 (11)
C11A	0.0273 (14)	0.0299 (15)	0.0303 (14)	−0.0005 (12)	0.0014 (11)	−0.0052 (12)
C12A	0.0277 (14)	0.0214 (13)	0.0294 (14)	0.0026 (11)	0.0045 (11)	−0.0054 (11)
C13A	0.0337 (15)	0.0235 (14)	0.0315 (15)	−0.0045 (12)	−0.0012 (12)	−0.0026 (11)
C14A	0.0269 (14)	0.0380 (17)	0.0367 (16)	−0.0042 (13)	−0.0002 (12)	0.0035 (13)
C15A	0.0177 (12)	0.0192 (12)	0.0217 (12)	−0.0005 (10)	0.0004 (9)	0.0022 (10)
C16A	0.0266 (13)	0.0212 (13)	0.0292 (14)	0.0026 (11)	0.0007 (11)	−0.0024 (11)
C17A	0.0232 (13)	0.0206 (13)	0.0388 (16)	0.0042 (11)	0.0010 (11)	0.0038 (11)
C18A	0.0203 (13)	0.0281 (14)	0.0297 (14)	−0.0021 (11)	−0.0012 (11)	0.0095 (11)
C19A	0.0257 (13)	0.0297 (14)	0.0235 (13)	−0.0017 (11)	0.0039 (11)	0.0033 (11)
C20A	0.0204 (12)	0.0216 (13)	0.0253 (13)	0.0019 (10)	0.0059 (10)	0.0009 (10)
C21A	0.0221 (12)	0.0227 (13)	0.0205 (12)	0.0006 (10)	0.0026 (10)	−0.0055 (10)
C22A	0.0244 (13)	0.0266 (14)	0.0275 (14)	0.0021 (11)	0.0035 (11)	−0.0011 (11)
C23A	0.0318 (15)	0.0294 (15)	0.0369 (16)	−0.0001 (12)	0.0105 (12)	0.0019 (12)
C24A	0.0273 (14)	0.0362 (16)	0.0364 (16)	−0.0063 (12)	0.0078 (12)	−0.0089 (13)
C25A	0.0226 (14)	0.0458 (19)	0.0436 (18)	0.0012 (13)	−0.0062 (13)	−0.0012 (15)
C26A	0.0282 (15)	0.0342 (16)	0.0349 (16)	0.0012 (12)	−0.0022 (12)	0.0031 (13)

### Bis(N,N-diallyldithiocarbamato-κ2S,S')diphenyltin(IV) (II) Geometric parameters (Å, º)

**Table:**

Sn1—C15	2.159 (3)	Sn2—C21A	2.170 (3)
Sn1—C21	2.174 (2)	Sn2—C15A	2.173 (2)
Sn1—S1	2.5501 (6)	Sn2—S1A	2.5585 (7)
Sn1—S3	2.5726 (7)	Sn2—S3A	2.5700 (6)
Sn1—S4	2.6754 (6)	Sn2—S4A	2.6750 (6)
Sn1—S2	2.7393 (7)	Sn2—S2A	2.7664 (7)
S1—C1	1.742 (3)	S1A—C1A	1.740 (3)
S2—C1	1.710 (3)	S2A—C1A	1.704 (3)
S3—C8	1.738 (3)	S3A—C8A	1.733 (3)
S4—C8	1.715 (3)	S4A—C8A	1.716 (3)
N1—C1	1.326 (3)	N1A—C1A	1.328 (4)
N1—C2	1.465 (3)	N1A—C5A	1.477 (4)
N1—C5	1.481 (3)	N1A—C2A	1.480 (3)
N2—C8	1.318 (3)	N2A—C8A	1.327 (3)
N2—C12	1.477 (3)	N2A—C9A	1.465 (3)
N2—C9	1.486 (3)	N2A—C12A	1.466 (3)
C2—C3	1.500 (4)	C2A—C3A	1.496 (5)
C2—H2A	0.9900	C2A—H2A1	0.9900
C2—H2B	0.9900	C2A—H2A2	0.9900
C3—C4	1.317 (4)	C3A—C4A	1.320 (5)
C3—H3	0.9500	C3A—H3A	0.9500
C4—H4A	0.9500	C4A—H4A1	0.9500
C4—H4B	0.9500	C4A—H4A2	0.9500
C5—C6	1.498 (4)	C5A—C6A	1.484 (4)
C5—H5A	0.9900	C5A—H5A1	0.9900
C5—H5B	0.9900	C5A—H5A2	0.9900
C6—C7	1.314 (4)	C6A—C7A	1.317 (6)
C6—H6	0.9500	C6A—H6A	0.9500
C7—H7A	0.9500	C7A—H7A1	0.9500
C7—H7B	0.9500	C7A—H7A2	0.9500
C9—C10	1.496 (4)	C9A—C10A	1.506 (4)
C9—H9A	0.9900	C9A—H9A1	0.9900
C9—H9B	0.9900	C9A—H9A2	0.9900
C10—C11	1.305 (5)	C10A—C11A	1.314 (4)
C10—H10	0.9500	C10A—H10A	0.9500
C11—H11A	0.9500	C11A—H11C	0.9500
C11—H11B	0.9500	C11A—H11D	0.9500
C12—C13	1.496 (5)	C12A—C13A	1.501 (4)
C12—H12A	0.9900	C12A—H12C	0.9900
C12—H12B	0.9900	C12A—H12D	0.9900
C13—C14	1.308 (5)	C13A—C14A	1.311 (4)
C13—H13	0.9500	C13A—H13A	0.9500
C14—H14A	0.9500	C14A—H14C	0.9500
C14—H14B	0.9500	C14A—H14D	0.9500
C15—C20	1.392 (4)	C15A—C16A	1.393 (4)
C15—C16	1.396 (4)	C15A—C20A	1.397 (4)
C16—C17	1.390 (4)	C16A—C17A	1.393 (4)
C16—H16	0.9500	C16A—H16A	0.9500
C17—C18	1.382 (4)	C17A—C18A	1.382 (4)
C17—H17	0.9500	C17A—H17A	0.9500
C18—C19	1.389 (4)	C18A—C19A	1.386 (4)
C18—H18	0.9500	C18A—H18A	0.9500
C19—C20	1.385 (4)	C19A—C20A	1.396 (4)
C19—H19	0.9500	C19A—H19A	0.9500
C20—H20	0.9500	C20A—H20A	0.9500
C21—C26	1.391 (4)	C21A—C22A	1.386 (4)
C21—C22	1.393 (4)	C21A—C26A	1.395 (4)
C22—C23	1.387 (4)	C22A—C23A	1.400 (4)
C22—H22	0.9500	C22A—H22A	0.9500
C23—C24	1.382 (4)	C23A—C24A	1.380 (4)
C23—H23	0.9500	C23A—H23A	0.9500
C24—C25	1.384 (4)	C24A—C25A	1.373 (5)
C24—H24	0.9500	C24A—H24A	0.9500
C25—C26	1.394 (4)	C25A—C26A	1.396 (4)
C25—H25	0.9500	C25A—H25A	0.9500
C26—H26	0.9500	C26A—H26A	0.9500
			
C15—Sn1—C21	99.84 (9)	C21A—Sn2—C15A	103.34 (9)
C15—Sn1—S1	104.01 (7)	C21A—Sn2—S1A	96.36 (7)
C21—Sn1—S1	92.77 (7)	C15A—Sn2—S1A	101.31 (7)
C15—Sn1—S3	94.80 (7)	C21A—Sn2—S3A	105.20 (7)
C21—Sn1—S3	101.09 (7)	C15A—Sn2—S3A	95.16 (7)
S1—Sn1—S3	154.38 (2)	S1A—Sn2—S3A	149.00 (2)
C15—Sn1—S4	160.59 (7)	C21A—Sn2—S4A	92.74 (7)
C21—Sn1—S4	92.52 (7)	C15A—Sn2—S4A	159.91 (7)
S1—Sn1—S4	90.18 (2)	S1A—Sn2—S4A	88.58 (2)
S3—Sn1—S4	67.97 (2)	S3A—Sn2—S4A	68.69 (2)
C15—Sn1—S2	91.81 (7)	C21A—Sn2—S2A	161.40 (7)
C21—Sn1—S2	159.42 (7)	C15A—Sn2—S2A	88.92 (7)
S1—Sn1—S2	67.824 (19)	S1A—Sn2—S2A	67.18 (2)
S3—Sn1—S2	94.71 (2)	S3A—Sn2—S2A	87.26 (2)
S4—Sn1—S2	81.21 (2)	S4A—Sn2—S2A	78.76 (2)
C1—S1—Sn1	89.92 (8)	C1A—S1A—Sn2	90.15 (9)
C1—S2—Sn1	84.47 (9)	C1A—S2A—Sn2	84.16 (9)
C8—S3—Sn1	89.22 (9)	C8A—S3A—Sn2	87.98 (9)
C8—S4—Sn1	86.37 (9)	C8A—S4A—Sn2	84.96 (9)
C1—N1—C2	122.5 (2)	C1A—N1A—C5A	122.0 (2)
C1—N1—C5	122.6 (2)	C1A—N1A—C2A	123.4 (2)
C2—N1—C5	114.9 (2)	C5A—N1A—C2A	114.5 (2)
C8—N2—C12	122.5 (2)	C8A—N2A—C9A	122.4 (2)
C8—N2—C9	122.6 (2)	C8A—N2A—C12A	122.1 (2)
C12—N2—C9	114.8 (2)	C9A—N2A—C12A	115.5 (2)
N1—C1—S2	123.62 (19)	N1A—C1A—S2A	122.7 (2)
N1—C1—S1	118.58 (19)	N1A—C1A—S1A	119.4 (2)
S2—C1—S1	117.77 (14)	S2A—C1A—S1A	117.94 (16)
N1—C2—C3	112.5 (2)	N1A—C2A—C3A	110.9 (2)
N1—C2—H2A	109.1	N1A—C2A—H2A1	109.5
C3—C2—H2A	109.1	C3A—C2A—H2A1	109.5
N1—C2—H2B	109.1	N1A—C2A—H2A2	109.5
C3—C2—H2B	109.1	C3A—C2A—H2A2	109.5
H2A—C2—H2B	107.8	H2A1—C2A—H2A2	108.1
C4—C3—C2	126.2 (3)	C4A—C3A—C2A	123.2 (3)
C4—C3—H3	116.9	C4A—C3A—H3A	118.4
C2—C3—H3	116.9	C2A—C3A—H3A	118.4
C3—C4—H4A	120.0	C3A—C4A—H4A1	120.0
C3—C4—H4B	120.0	C3A—C4A—H4A2	120.0
H4A—C4—H4B	120.0	H4A1—C4A—H4A2	120.0
N1—C5—C6	111.3 (2)	N1A—C5A—C6A	110.9 (2)
N1—C5—H5A	109.4	N1A—C5A—H5A1	109.5
C6—C5—H5A	109.4	C6A—C5A—H5A1	109.5
N1—C5—H5B	109.4	N1A—C5A—H5A2	109.5
C6—C5—H5B	109.4	C6A—C5A—H5A2	109.5
H5A—C5—H5B	108.0	H5A1—C5A—H5A2	108.0
C7—C6—C5	124.0 (3)	C7A—C6A—C5A	122.9 (4)
C7—C6—H6	118.0	C7A—C6A—H6A	118.5
C5—C6—H6	118.0	C5A—C6A—H6A	118.5
C6—C7—H7A	120.0	C6A—C7A—H7A1	120.0
C6—C7—H7B	120.0	C6A—C7A—H7A2	120.0
H7A—C7—H7B	120.0	H7A1—C7A—H7A2	120.0
N2—C8—S4	122.3 (2)	N2A—C8A—S4A	121.67 (19)
N2—C8—S3	121.3 (2)	N2A—C8A—S3A	120.11 (19)
S4—C8—S3	116.40 (15)	S4A—C8A—S3A	118.21 (15)
N2—C9—C10	109.6 (2)	N2A—C9A—C10A	113.2 (2)
N2—C9—H9A	109.8	N2A—C9A—H9A1	108.9
C10—C9—H9A	109.8	C10A—C9A—H9A1	108.9
N2—C9—H9B	109.8	N2A—C9A—H9A2	108.9
C10—C9—H9B	109.8	C10A—C9A—H9A2	108.9
H9A—C9—H9B	108.2	H9A1—C9A—H9A2	107.7
C11—C10—C9	123.4 (3)	C11A—C10A—C9A	126.1 (3)
C11—C10—H10	118.3	C11A—C10A—H10A	117.0
C9—C10—H10	118.3	C9A—C10A—H10A	117.0
C10—C11—H11A	120.0	C10A—C11A—H11C	120.0
C10—C11—H11B	120.0	C10A—C11A—H11D	120.0
H11A—C11—H11B	120.0	H11C—C11A—H11D	120.0
N2—C12—C13	110.8 (2)	N2A—C12A—C13A	112.3 (2)
N2—C12—H12A	109.5	N2A—C12A—H12C	109.1
C13—C12—H12A	109.5	C13A—C12A—H12C	109.1
N2—C12—H12B	109.5	N2A—C12A—H12D	109.1
C13—C12—H12B	109.5	C13A—C12A—H12D	109.1
H12A—C12—H12B	108.1	H12C—C12A—H12D	107.9
C14—C13—C12	123.8 (3)	C14A—C13A—C12A	126.5 (3)
C14—C13—H13	118.1	C14A—C13A—H13A	116.7
C12—C13—H13	118.1	C12A—C13A—H13A	116.7
C13—C14—H14A	120.0	C13A—C14A—H14C	120.0
C13—C14—H14B	120.0	C13A—C14A—H14D	120.0
H14A—C14—H14B	120.0	H14C—C14A—H14D	120.0
C20—C15—C16	118.6 (2)	C16A—C15A—C20A	118.3 (2)
C20—C15—Sn1	118.07 (19)	C16A—C15A—Sn2	120.81 (19)
C16—C15—Sn1	123.31 (19)	C20A—C15A—Sn2	120.91 (18)
C17—C16—C15	120.3 (2)	C17A—C16A—C15A	121.1 (3)
C17—C16—H16	119.8	C17A—C16A—H16A	119.5
C15—C16—H16	119.8	C15A—C16A—H16A	119.5
C18—C17—C16	120.4 (3)	C18A—C17A—C16A	119.8 (3)
C18—C17—H17	119.8	C18A—C17A—H17A	120.1
C16—C17—H17	119.8	C16A—C17A—H17A	120.1
C19—C18—C17	119.9 (3)	C17A—C18A—C19A	120.3 (2)
C19—C18—H18	120.1	C17A—C18A—H18A	119.9
C17—C18—H18	120.1	C19A—C18A—H18A	119.9
C18—C19—C20	119.7 (2)	C18A—C19A—C20A	119.7 (3)
C18—C19—H19	120.2	C18A—C19A—H19A	120.2
C20—C19—H19	120.2	C20A—C19A—H19A	120.2
C19—C20—C15	121.2 (2)	C15A—C20A—C19A	120.9 (2)
C19—C20—H20	119.4	C15A—C20A—H20A	119.6
C15—C20—H20	119.4	C19A—C20A—H20A	119.6
C26—C21—C22	117.6 (2)	C22A—C21A—C26A	118.0 (3)
C26—C21—Sn1	123.63 (19)	C22A—C21A—Sn2	118.00 (19)
C22—C21—Sn1	118.80 (19)	C26A—C21A—Sn2	124.0 (2)
C23—C22—C21	121.4 (3)	C21A—C22A—C23A	121.3 (3)
C23—C22—H22	119.3	C21A—C22A—H22A	119.4
C21—C22—H22	119.3	C23A—C22A—H22A	119.4
C24—C23—C22	120.2 (3)	C24A—C23A—C22A	119.7 (3)
C24—C23—H23	119.9	C24A—C23A—H23A	120.1
C22—C23—H23	119.9	C22A—C23A—H23A	120.1
C23—C24—C25	119.4 (3)	C25A—C24A—C23A	119.7 (3)
C23—C24—H24	120.3	C25A—C24A—H24A	120.1
C25—C24—H24	120.3	C23A—C24A—H24A	120.1
C24—C25—C26	120.1 (3)	C24A—C25A—C26A	120.7 (3)
C24—C25—H25	120.0	C24A—C25A—H25A	119.7
C26—C25—H25	120.0	C26A—C25A—H25A	119.7
C21—C26—C25	121.3 (3)	C21A—C26A—C25A	120.5 (3)
C21—C26—H26	119.4	C21A—C26A—H26A	119.8
C25—C26—H26	119.4	C25A—C26A—H26A	119.8
			
C2—N1—C1—S2	0.1 (3)	C5A—N1A—C1A—S2A	4.7 (4)
C5—N1—C1—S2	179.23 (18)	C2A—N1A—C1A—S2A	−175.2 (2)
C2—N1—C1—S1	−178.18 (18)	C5A—N1A—C1A—S1A	−175.4 (2)
C5—N1—C1—S1	1.0 (3)	C2A—N1A—C1A—S1A	4.8 (4)
Sn1—S2—C1—N1	−177.4 (2)	Sn2—S2A—C1A—N1A	172.9 (2)
Sn1—S2—C1—S1	0.89 (13)	Sn2—S2A—C1A—S1A	−7.00 (14)
Sn1—S1—C1—N1	177.43 (19)	Sn2—S1A—C1A—N1A	−172.4 (2)
Sn1—S1—C1—S2	−0.95 (13)	Sn2—S1A—C1A—S2A	7.53 (15)
C1—N1—C2—C3	−95.8 (3)	C1A—N1A—C2A—C3A	−98.2 (3)
C5—N1—C2—C3	85.0 (3)	C5A—N1A—C2A—C3A	81.9 (3)
N1—C2—C3—C4	12.5 (4)	N1A—C2A—C3A—C4A	−105.2 (3)
C1—N1—C5—C6	−91.2 (3)	C1A—N1A—C5A—C6A	−97.9 (3)
C2—N1—C5—C6	88.0 (3)	C2A—N1A—C5A—C6A	82.0 (3)
N1—C5—C6—C7	−122.3 (3)	N1A—C5A—C6A—C7A	−114.6 (4)
C12—N2—C8—S4	179.3 (2)	C9A—N2A—C8A—S4A	2.4 (4)
C9—N2—C8—S4	1.2 (4)	C12A—N2A—C8A—S4A	−173.87 (19)
C12—N2—C8—S3	−0.7 (4)	C9A—N2A—C8A—S3A	−178.66 (19)
C9—N2—C8—S3	−178.79 (19)	C12A—N2A—C8A—S3A	5.1 (3)
Sn1—S4—C8—N2	178.1 (2)	Sn2—S4A—C8A—N2A	175.2 (2)
Sn1—S4—C8—S3	−1.92 (13)	Sn2—S4A—C8A—S3A	−3.74 (14)
Sn1—S3—C8—N2	−178.1 (2)	Sn2—S3A—C8A—N2A	−175.1 (2)
Sn1—S3—C8—S4	1.99 (14)	Sn2—S3A—C8A—S4A	3.88 (15)
C8—N2—C9—C10	101.8 (3)	C8A—N2A—C9A—C10A	88.1 (3)
C12—N2—C9—C10	−76.4 (3)	C12A—N2A—C9A—C10A	−95.3 (3)
N2—C9—C10—C11	105.3 (3)	N2A—C9A—C10A—C11A	−9.9 (4)
C8—N2—C12—C13	104.1 (3)	C8A—N2A—C12A—C13A	90.9 (3)
C9—N2—C12—C13	−77.6 (3)	C9A—N2A—C12A—C13A	−85.6 (3)
N2—C12—C13—C14	110.9 (4)	N2A—C12A—C13A—C14A	−13.3 (4)
C20—C15—C16—C17	−0.4 (4)	C20A—C15A—C16A—C17A	0.9 (4)
Sn1—C15—C16—C17	176.7 (2)	Sn2—C15A—C16A—C17A	−177.9 (2)
C15—C16—C17—C18	0.9 (4)	C15A—C16A—C17A—C18A	−0.7 (4)
C16—C17—C18—C19	−0.7 (4)	C16A—C17A—C18A—C19A	−0.1 (4)
C17—C18—C19—C20	0.0 (4)	C17A—C18A—C19A—C20A	0.7 (4)
C18—C19—C20—C15	0.6 (4)	C16A—C15A—C20A—C19A	−0.3 (4)
C16—C15—C20—C19	−0.4 (4)	Sn2—C15A—C20A—C19A	178.47 (19)
Sn1—C15—C20—C19	−177.60 (19)	C18A—C19A—C20A—C15A	−0.5 (4)
C26—C21—C22—C23	−0.7 (4)	C26A—C21A—C22A—C23A	−1.2 (4)
Sn1—C21—C22—C23	180.0 (2)	Sn2—C21A—C22A—C23A	179.5 (2)
C21—C22—C23—C24	−0.2 (4)	C21A—C22A—C23A—C24A	1.1 (4)
C22—C23—C24—C25	0.5 (4)	C22A—C23A—C24A—C25A	−0.4 (4)
C23—C24—C25—C26	0.1 (4)	C23A—C24A—C25A—C26A	−0.1 (5)
C22—C21—C26—C25	1.2 (4)	C22A—C21A—C26A—C25A	0.7 (4)
Sn1—C21—C26—C25	−179.5 (2)	Sn2—C21A—C26A—C25A	180.0 (2)
C24—C25—C26—C21	−0.9 (4)	C24A—C25A—C26A—C21A	−0.1 (5)

### Bis(N,N-diallyldithiocarbamato-κ2S,S')diphenyltin(IV) (II) Hydrogen-bond geometry (Å, º)

*Cg*1 and *Cg*2 are the centroids of the (C15*A*–C20*A*) and (C21*A*–C26*A*) rings, respectively.

**Table:**

*D*—H···*A*	*D*—H	H···*A*	*D*···*A*	*D*—H···*A*
C7—H7*A*···*Cg*1^i^	0.95	2.83	3.774 (3)	172
C7—H7*B*···*Cg*2^ii^	0.95	2.92	3.582 (3)	128

Symmetry codes: (i) −*x*+2, −*y*+1, −*z*+1; (ii) −*x*+1, −*y*+1, −*z*+1.
